# L-arginine in patients with spinocerebellar ataxia type 6: a multicentre, randomised, double-blind, placebo-controlled, phase 2 trial

**DOI:** 10.1016/j.eclinm.2024.102952

**Published:** 2024-11-25

**Authors:** Tomohiko Ishihara, Masayoshi Tada, Yoshitomi Kanemitsu, Yuji Takahashi, Kinya Ishikawa, Kensuke Ikenaka, Makito Hirano, Takanori Yokota, Eiko N. Minakawa, Katsuhisa Saito, Yoshitaka Nagai, Osamu Onodera

**Affiliations:** a Department of Neurology, Brain Research Institute, Niigata University, Niigata, Japan; b Advanced Treatment of Neurological Diseases Branch, Endowed Research Branch, Brain Research Institute, Niigata University, Niigata, Japan; c Department of Neurology, Niigata City General Hospital, Niigata, Japan; d Clinical and Translational Research Center, Niigata University Medical and Dental Hospital, Niigata, Japan; e Department of Neurology, National Center Hospital, National Center of Neurology and Psychiatry, Tokyo, Japan; f Department of Neurology and Neurological Science, Graduate School of Medical and Dental Sciences, Institute of Science Tokyo, Tokyo, Japan; g Department of Neurology, Osaka University Graduate School of Medicine, Suita, Japan; h Department of Neurology, Kindai University Faculty of Medicine, Osaka, Japan; i Department of Neurophysiology, National Institute of Neuroscience, National Center of Neurology and Psychiatry, Tokyo, Japan; j Department of Medical Innovation, Osaka University Graduate School of Medicine, Suita, Japan; k Life Science Research Institute, Kindai University Faculty of Medicine, Osaka, Japan; l Neurotherapeutics, Osaka University Graduate School of Medicine, Suita, Japan

**Keywords:** Spinocerebellar ataxia type 6, L-arginine, Assessment and rating of ataxia, Randomized placebo-controlled trial

## Abstract

**Background:**

Therapeutic advancements for the polyglutamine diseases, particularly spinocerebellar degeneration, are eagerly awaited. We evaluated the safety, tolerability, and therapeutic effects of L-arginine, which inhibits the conformational change and aggregation of polyglutamine proteins, in patients with spinocerebellar ataxia type 6 (SCA6).

**Methods:**

A multicenter, randomized, double-blind, placebo-controlled phase 2 trial (clinical trial ID: AJA030-002, registration number: jRCT2031200135) was performed on 40 genetically confirmed SCA6 patients enrolled between September 1, 2020, and September 30, 2021. The main inclusion criteria were as follows: SCA6 diagnosed by genetic testing, 20 years of age or older, Scale for the Assessment and Rating of Ataxia (SARA) “walking” score of at least one point, and SARA “total” score of at least 10 points, and ability to walk 10 m or more with or without an assistive device. The investigational drug was administered orally at 0.50 g/kg/day (L-arginine group: L-arginine 0.38 g/kg/day; placebo group: L-arginine 0.0 g/kg/day) for 48 weeks. Subjects who consented to participate were assigned a subject identification code, and were allocated 1:1 to the L-arginine or the placebo group, according to a predetermined allocation chart. The primary efficacy endpoint was change in total Scale for the Assessment and Rating of Ataxia (SARA) score from baseline to 48 weeks. The secondary endpoints were 1) SARA walking + standing score, 2) each of the eight SARA scales at 0, 4, 8, 16, 24, 32, 40, and 48 weeks, and 3) TUGT, BDI-II, CGI, PGI-I, and SF-8.

**Findings:**

Forty patients received the investigational drug, and 37 completed the study (L-arginine group: 18; placebo group: 19). The mean medication adherence rate was 97.2% in the l-arginine group. Regarding the primary endpoint, the difference between the L-arginine group and the placebo group was −1.52 (95% CI: −3.10 to 0.06, *P =* 0.0582). As the secondary endpoints, the change of SARA total score from baseline was greater in the L-arginine group than in the placebo group at all assessment time points, but the differences were not significant. Two serious (required hospitalization) adverse reactions occurred in the L-arginine group, including one case of pneumonia (severe, death) and one case of abnormal liver function (moderate, recovery).

**Interpretation:**

L-arginine treatment resulted in an improvement tendency in SARA total score of SCA6 patients. Our results suggest that a phase 3 study of L-arginine for SCA6, with a 48-week observation period and change in total SARA score as the primary endpoint, may be feasible for further analyzing the therapeutic effect of L-arginine. However, careful consideration of statistical power and sample size is necessary.

**Funding:**

Japan Agency for Medical Research and Development and Health Labour Sciences Research Grant, Japan.

**Research in context**

**Evidence before this study**

Spinocerebellar degeneration (SCD) refers to a group of intractable neurodegenerative disorders primarily characterized by ataxia. In Japan, most hereditary SCDs, including spinocerebellar ataxia types 1, 2, 3, 6, 7, and 17 (SCA1, 2, 3, 6, 7, and 17), and dentatorubral-pallidoluysian atrophy, are polyglutamine diseases. In these molecular pathogeneses, the expanded polyglutamine tract in mutant proteins undergo conformational changes, becoming cytotoxic. Recently, we discovered that L-arginine, known as a chemical chaperone, can stabilizes the structure of polyglutamine protein, inhibiting their conformational change and aggregation in animal models. In this study, we assessed the efficacy of L-arginine in patients with SCA6 in a Randomized Controlled Trial (RCT). The investigational drug was administered orally at 0.50 g/kg/day (L-arginine group: L-arginine 0.38 g/kg/day; placebo group: L-arginine 0.0 g/kg/day) for 48 weeks.

We searched PubMed with the terms “spinocerebellar ataxia type 6” OR “SCA6,” AND filtered the article type with “Randomized Controlled Trial” for articles published in English up to March 31, 2024. Two RCTs were identified. One investigated the effect of 4 weeks of home rehabilitation in SCA6 patients, showing no statistically significant effect. The other trial evaluated the efficacy of rovatirelin for 24 or 28 weeks in patients with SCA6, SCA31, or cortical cerebellar atrophy. This trial also failed to show a statistically significant effect. However, an important finding in the analysis was a greater placebo effect in patients with milder disease.

**Added value of this study**

The difference between the l-arginine and placebo groups in change in Scale for the Assessment and Rating of Ataxia (SARA) total score after 48 weeks was −1.52 (95% CI: −3.101 to 0.055, *P* = 0.0582). The change from baseline was greater in the l-arginine group than in the placebo group at all assessment time points. A unique feature of this trial was the exclusion of mild cases in the entry criteria. This might exclude the placebo effect in mild cases seen in previous studies.

**Implications of all the available evidence**

Administration of l-arginine may have clinical benefits for SCA6 patients. Since l-arginine is already approved in Japan for urea cycle disorders, it offers advantages in terms of drug repositioning. A larger study may provide statistical evidence supporting the efficacy of l-arginine in this patient population.

## Introduction

Spinocerebellar degeneration (SCD) is a group of neurodegenerative disorders with core symptoms of ataxia. SCD is classified as either sporadic or hereditary. Most hereditary SCD cases in Japan are classified as the polyglutamine diseases, such as spinocerebellar ataxia types 1, 2, 3, 6, 7, and 17 (SCA1, 2, 3, 6, 7, and 17), and dentatorubral-pallidoluysian atrophy.^1^ The polyglutamine diseases are caused by the abnormal expansion of a CAG repeat sequence encoding a polyglutamine tract within the causative gene.^2^ There is a correlation between the length of the expanded CAG repeat and clinical symptoms.^3^ However, the pathological length of CAG repeats and the range of clinical symptom severity vary between the diseases. In the molecular pathogenesis of the polyglutamine diseases, mutant proteins with expanded polyglutamine tracts undergo conformational changes and become toxic to cells.^4^, ^5^, ^6^, ^7^ Therefore, various therapeutic strategies to reduce the amount of mutant protein and to prevent the toxic conformational changes have been proposed to date.^8^

Methods to reduce the levels of pathological proteins using antisense oligonucleotides and small interfering RNA have already been used to treat various diseases, such as hereditary transthyretin amyloidosis,^9^ acute hepatic porphyria,^10^ and familial amyotrophic lateral sclerosis type 1.^11^ Preclinical studies of such methods have been performed for various polyglutamine diseases, including cell culture and animal models of SCA2 and SCA3, while clinical trials have been conducted for Huntington's disease (HD).^12^, ^13^, ^14^, ^15^ However, as was problematic for HD disease therapy development, when using strategies that suppress disease-causing protein expression, the negative effects of suppressing the normal function of the target protein must be considered.^16^ The causative gene for SCA6, a polyglutamine disease, is the voltage-gated calcium channel. Loss or dysfunction of this protein leads to the development of neurological diseases.^17^^,^^18^ Therefore, a therapeutic strategy other than reducing its protein expression is required for this disease.

The development of chemical chaperones, which are small-molecule compounds that interfere with the conformational changes that occur in mutant proteins, has been attempted.^19^ We found that the l-arginine, which is known to act as a chemical chaperone, stabilizes the structure of polyglutamine proteins, and thereby inhibits their conformational change and suppresses their aggregation.^20^ Furthermore, we demonstrated that in the SCA1 mouse model, oral administration of arginine resulted in a dose-dependent increase in arginine levels in their serum and brain, and reduced polyglutamine-positive inclusion bodies in the brain tissues.^20^ Furthermore, we showed that l-arginine administration partly resolved the symptoms of two different polyglutamine disease mouse models.^20^ l-arginine has been shown to be safe and well tolerated in humans, and has been used as a drug for congenital urea cycle abnormalities, and mitochondrial myopathy, encephalopathy, lactic acidosis, and stroke-like episodes (MELAS).^21^^,^^22^

Here, we conducted a Phase 2 pilot study to investigate the safety and tolerability of l-arginine, and evaluate its therapeutic effects in patients with SCA6. SCA6 was selected in this study because as explained above, therapies that suppress expression levels of the target protein need to be avoided. Furthermore, the symptoms and progression of the disease are relatively uniform compared with the other polyglutamine diseases owing to the narrow range of CAG repeat lengths present among patients, making it easier to match the clinical conditions between patients in the l-arginine and placebo groups.^3^^,^^18^

## Methods

### Study design and participants

A multicenter, randomized, double-blind, placebo-controlled phase 2 trial (clinical trial ID: AJA030-002, registration number: jRCT2031200135) was conducted as an exploratory study on the effect size, safety, and tolerability of l-arginine in SCA6 patients. The target number of patients was set at 20 in each group, for a total of 40 patients. This was set as the minimum number of patients required in consideration of feasibility. Subjects from five hospitals in Japan were enrolled between September 1, 2020, and September 30, 2021.

Patient selection criteria were as follows: 1) SCA6 diagnosed by genetic testing, 2) 20 years of age or older at the time of consent, 3) written consent for participation in the study obtained from the patient, 4) Scale for the Assessment and Rating of Ataxia (SARA) “walking” score of at least one point, and SARA “total” score of at least 10 points, and 5) ability to walk 10 m or more with or without an assistive device. Among these, 4) and 5) are particularly noteworthy. In clinical trials of spinocerebellar degeneration, a substantial placebo effect has been reported to occur,^23^ and the effect is particularly large in patients with mild symptoms.^24^ Therefore, we decided to include patients with a specific range of disease severity in this trial. In addition, we did not include patients with more than a certain level of severity, that is, those who were unable to walk, because we considered the possibility that symptom progression would reach a plateau. Patients with dementia (Mini-Mental State Examination (MMSE) score ≤23) and psychiatric complications, including untreated depression (Beck Depression Inventory score ≥21), were also excluded.

### Randomization and masking

Forty subjects who consented to participate in the study were assigned a subject identification code, and were allocated 1:1 to the l-arginine or the placebo group, according to a predetermined study drug allocation chart. All patients and personnel, including the investigators and medical staff at the study sites, were blinded to the treatment allocation. The investigational drug was packaged in individual aluminum packages (3 g each) and was not identifiable by appearance. In addition, the results of blood arginine levels were kept at an external laboratory until unblinding was completed.

Random allocation of investigational drugs and maintenance of blindness were performed according to the protocol. The investigational drug randomization allocation list was prepared by an assignment manager (Accerise Inc. Contract Research Organization). Each subject who consented to participate in the study was assigned a subject identification code in the order explained, and the assignment manager assigned the investigational drug according to the randomization allocation list. To maintain blinding, the randomization allocation list was sealed in an opaque envelope and secured by the assignment manager. The study coordinator maintained emergency key codes for adverse events. After all participants had been dosed and evaluated and after data fixation was completed, a meeting was held to open the allocation chart and disclose the results of the drug allocation. At that time, it was confirmed that the investigational drug allocation table and emergency key codes were not opened.

### Procedures

The active drug was ARGI-U combination granules (EA Pharma Inc.), a combination of l-arginine hydrochloride and l-arginine. The amount of investigational drug to be taken internally, whether active drug or placebo, was 0.50 g/kg/day. The amount of l-arginine in 1 g of the investigational drug was 0.76 g for the active drug and 0 g for the placebo. Thus, the dose of l-arginine to be taken was 0.38 g/kg/day for the active drug group and 0 g for the placebo group. The investigational drug was administered orally three times daily for 48 weeks. The dose was adjusted every 3 g according to body weight, with a maximum of 33 g/day (Supplementary Table S1). No investigational drug was administered during the 4-week follow-up period from weeks 48–52. Patients were permitted to take their regular medications (including taltirelin hydrate and protirelin tartrate hydrate) during the study period. However, the dose of all regular medications were kept constant for four weeks prior to the start of administration of the investigational drug.

For safety evaluation, vital signs (temperature, blood pressure, and pulse), body weight, adverse events (AEs), and hematology, blood biochemistry, and urinalysis data were obtained before enrollment, on the first day of treatment, and after 4, 8, 16, 24, 32, 40, 48, and 52 weeks of treatment. Medication compliance was confirmed based on each patient's medication logbook and number of empty investigational drug packages.

SARA (Japanese version) scores^25^^,^^26^ and Clinical Global Impression-Severity (CGI-S)^27^ were evaluated on the day of treatment initiation, and at 4, 8, 16, 24, 32, 40, 48, and 52 weeks thereafter. The Timed Up and Go test (TUGT) and Beck Depression Inventory-II (BDI-II)^28^ were performed on the first day of treatment and at 48 and 52 weeks, and the Clinical Global Impression-Improvement (CGI-I), Patient Global Impression-Improvement (PGI-I),^27^ and Short-Form 8 (SF-8)^29^ were evaluated at 4, 8,16, 24, 32, 40, 48, and 52 weeks. Blood arginine levels were also measured at the beginning of treatment and at 4, 24, 48, and 52 weeks. For the measurement of blood levels, samples were collected at least 6 h after investigational drug dosing.

SARA evaluations were performed at each institution, by a neurologist separate from the physician prescribing the investigational drug. The evaluating physicians received video training on the SARA assessment procedure. The TUGT measures the time taken to rise from a chair, go around a landmark 3 m away, and sit in the chair again. The test was performed once at normal walking speed and once at maximum walking speed, and the maximum walking speed was recorded.

### Outcomes

The primary efficacy endpoint was the change in SARA total score from baseline to 48 weeks. The secondary efficacy endpoints were SARA total score, standing and gait scores, other individual SARA scores, and TUGT, BDI-II, CGI-S, CGI-I, PGI-I, and SF-8 scores at each evaluation time point.

Safety was assessed at each visit using vital signs (temperature, blood pressure, and pulse), confirmation of AEs, and hematology, blood biochemistry, and urine analysis. AEs were evaluated regarding their date of onset, severity (mild, moderate, or severe), causal association with the investigational drug, treatment, and outcome. Adverse reactions (ARs) were defined as AEs for which a causal association with the investigational drug could not be ruled out.

### Statistical analyses

Statistical analyses were performed using a predetermined statistical analysis protocol. Analysis populations were defined as follows: 1) Full analysis set (FAS): analysis set including subjects who were administered at least one dose of the investigational drug and have at least one post-baseline efficacy measure. 2) Per protocol set (PPS): analysis set conforming to the study protocol. In accordance with the predetermined statistical analysis protocol, the primary endpoint was analyzed using the FAS. The stability of the analysis results was also evaluated using the PPS. The safety analysis set (SAS) excluded subjects who were not administered the investigational drug and those for whom no safety observation was performed after administration of the investigational drug. Statistical analyses were performed using SAS ver. 9.4 software.

The study was conducted at five similarly sized centers in Japan. Each center enrolled either 5 or 10 patients, so stratified randomization by facility was not performed.

### Ethics

This study was implemented based on the ethics policies of Good Clinical Practice and the Declaration of Helsinki. Implementation of the study was approved by the institutional review board of each institution before the start of the study. Written informed consent was obtained from all the patients. The trial was filed and registered at the Pharmaceuticals and Medical Devices Agency in Japan (registration number: jRCT2031200135).

### Role of the funding source

The funder of the study had no role in the study design, data collection, data analysis, data interpretation, or writing of the report.

## Results

### Patient population

**Fig. 1:**
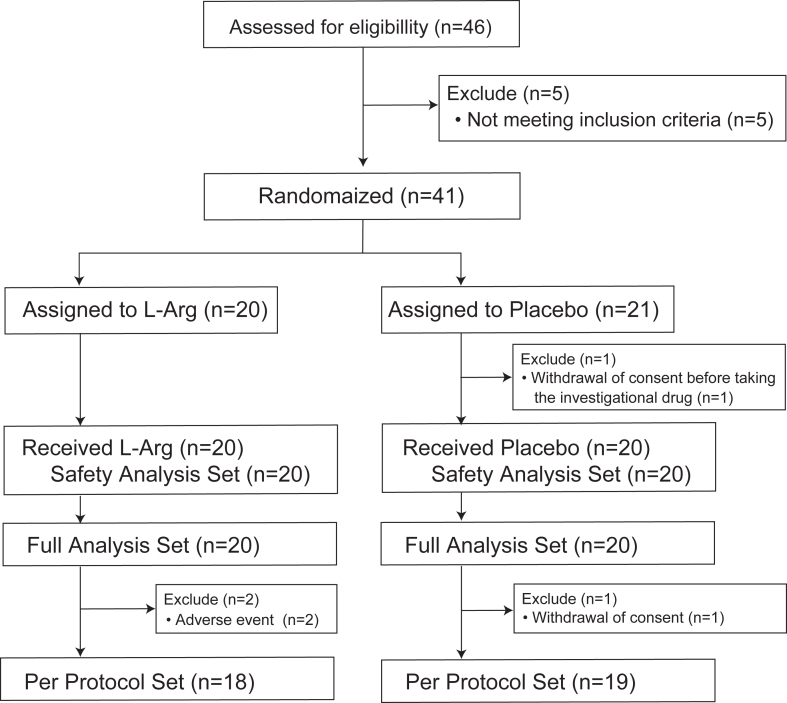
**Flow chart of patients included in the AJA030-002 study**. L-Arg, L-Arginine.

The characteristics of the participants in this clinical trial are shown in Fig. 1. Of the enrolled subjects, 41 met the study entry criteria, one of whom withdrew consent before taking the investigational drug. Forty patients received the investigational drug and 37 completed the study (18 in the l-arginine group and 19 in the placebo group). Patients in this trial weighing more than 70 kg had a lower relative oral dose (Supplementary Table S1): seven patients in the active drug group and four patients in the placebo group weighed more than 70 kg. The study population included 40 patients in the FAS (20 in the l-arginine group and 20 in the placebo group), 37 patients in the PPS (18 in the l-arginine group and 19 in the placebo group), and 40 patients in the SAS (20 in the l-arginine group and 20 in the placebo group).

Table 1 shows the characteristics of the patient groups. Supplementary Table S2 shows the medical history, and Supplementary Table S3 shows the complications at the time of obtaining consent. The age at consent to participate was 66.3 ± 7.9 years (mean ± standard deviation [SD]) in the l-arginine group, and 63.6 ± 11.9 years (mean ± SD) in the placebo group. The total SARA score at baseline was 15.7 ± 3.4 (mean ± SD) (minimum: 10; maximum: 25.5) in the l-arginine group, and 14.8 ± 3.5 (mean ± SD) (minimum: 10.5; maximum: 20.5) in the placebo group (Table 1).

**Table 1:** Characteristics of each group.

	L-arginine	Placebo
	n = 20	n = 20
Sex		
Male	11 (55.0%)	11 (55.0%)
Female	9 (45.0%)	9 (45.0%)
Age at the time of consent to participate (Years)		
20–39	0 (0.0%)	1 (5.0%)
40–50	5 (25.0%)	5 (25.0%)
60–79	15 (75.0%)	14 (70.0%)
80-	0 (0.0%)	0 (0.0%)
Mean ± SD	66.3 ± 7.9	63.6 ± 11.9
Median (Min, Max)	68.0 (47, 78)	67.5 (39, 79)
Weight (kg)		
Mean ± SD	62.9 ± 14.2	60.9 ± 11.8
Median (Min, Max)	62.2 (35.1, 85.6)	59.6 (41.8, 86.1)
SARA total score at time of consent to participate		
Mean ± SD	15.7 ± 3.4	14.8 ± 3.5
(Min, Max)	(10, 25.5)	(10.5, 20.5)
SARA walking score at the time of consent		
Mean ± SD	4.0 ± 1.2	3.7 ± 1.5
(Min, Max)	(3, 6)	(1, 6)
Medical history		
Absent	8 (40.0%)	7 (35.0%)
Present	12 (60.0%)	13 (65.0%)
Complication		
Absent	0 (0.0%)	2 (10.0%)
Present	20 (100.0%)	18 (90.0%)

SARA, Scale for the Assessment and Rating of Ataxia; SD, Standard deviation.

### Medication adherence rate

**Fig. 2:**
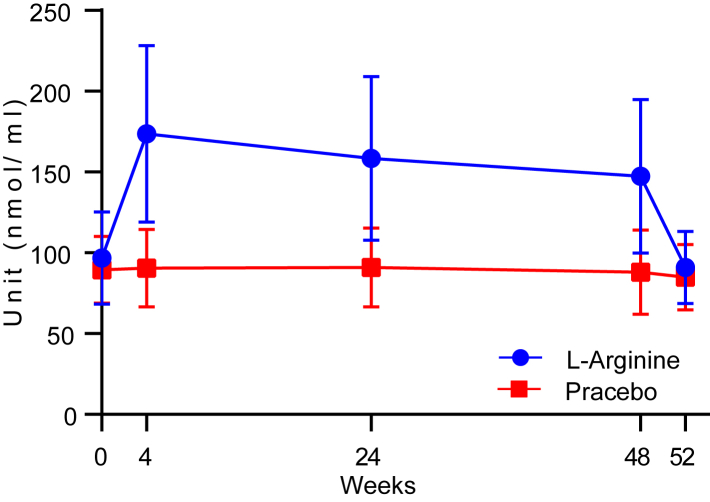
**Time-dependent changes in blood arginine levels in SCA6 patients**. Data are shown as the mean and SD. SCA6, spinocerebellar ataxia types 6.

Supplementary Table S4 shows the medication adherence rate of the 40 patients in SAS analysis. Good medication adherence rate (>80%) was observed in 20 patients (100.0%) in both the l-arginine and placebo groups. The mean medication adherence rate was 97.2% (Min 83.2, Max 99.9) in the l-arginine group and 98.8 (Min 90.5, Max 99.8) in the placebo group. Blood arginine levels increased steadily during the treatment period, and rapidly decreased to premedication levels at 52 weeks, and at four weeks after completion of treatment (Fig. 2).

### Safety

In the SAS analysis, 17 out of 20 (85.0%) patients in the l-arginine group had AEs (total: 64 cases) and 17 out of 20 (85.0%) patients in the placebo group had AEs (total: 66 cases) (Table 2, Supplementary Tables S5 and S6). Two patients (two event) in the l-arginine group and five patients (five event) in the placebo group experienced serious AEs (Table 2). Since inpatient treatment was required, these cases were considered as serious AE. The two serious AEs in the l-arginine group included one case of pneumonia (death) and one case of abnormal liver function (recovery). Pneumonia case: A fever of 37–38 °C developed after 8 weeks on study medication. Results of multiple tests for COVID-19 were negative. The patient was treated with antibiotics, but did not respond and died on day 15 of hospitalization. Abnormal liver function case: Eight weeks after beginning treatment with the study drug, blood tests showed elevated hepatobiliary enzymes, and the patient was urgently admitted to the hospital. Both Taltirelin Hydrate and the study drug were discontinued on the day of admission; hepatobiliary enzyme levels peaked two days later. The hepatic function improved, but the investigational drugs were not restarted. Patch tests were negative for both drugs. Five serious AEs were reported in the placebo group: (time of onset from start of study medication, outcome): COVID-19 infection (3 weeks, recovery), femoral fracture (3 days, recovery), subdural hematoma (4 weeks, recovery), wrist ligament injury (5 months, recovery), breast cancer (4 months, improvement). Regarding ARs, there were eight patients (40.0%; 12 cases) in the l-arginine group and five patients (25.0%; seven cases) in the placebo group (Table 2). The occurrence of ARs in the placebo group, primarily abdominal discomfort, may be attributed to the high oral dose of placebo (up to 33 g/day). This highlights the importance of considering potential side effects even with seemingly inert placebo substances when administered in large quantities.

**Table 2:** Adverse Events (AEs), adverse reactions (ARs).

	L-Arginine	Placebo
n	20	20
Any AE, no. of patients, n (%)	17 (85.0%)	17 (85.0%)
Any AE, no. of events	64	66
Serious AE, no. of patients, n (%)	2 (10.0%)	5 (25.0%)
Any AR, no. of patients, n (%)	8 (40.0%)	5 (25.0%)

AR event^a^	Events	Patients	%	Events	Patients	%
Abdominal discomfort	0	0	(0.0%)	4	3	(15.0%)
Abdominal distension	1	1	(5.0%)	1	1	(5.0%)
Ischemic colitis	0	0	(0.0%)	1	1	(5.0%)
Constipation	1	1	(5.0%)	0	0	(0.0%)
Diarrhea	1	1	(5.0%)	0	0	(0.0%)
Vomiting	1	1	(5.0%)	0	0	(0.0%)
Abnormal liver function	2	1	(5.0%)	0	0	(0.0%)
Pneumonia	1	1	(5.0%)	0	0	(0.0%)
Increased blood triglycerides	1	1	(5.0%)	0	0	(0.0%)
Positive urinary glucose	1	1	(5.0%)	0	0	(0.0%)
Increased glycohemoglobin	1	1	(5.0%)	0	0	(0.0%)
Weight increase	1	1	(5.0%)	0	0	(0.0%)
Positive urinary occult blood	1	1	(5.0%)	0	0	(0.0%)
Headache	0	0	(0.0%)	1	1	(5.0%)
Death, n (%)		1 (5.0%)			0 (0.0%)	
Severity of AE, no of events						
Mild		53			33	
Moderate		6			17	
Severe		1			2	

a Some patients have multiple AR events, so the total cases don't match the Any AR column.

### Efficacy

The primary endpoint was the change in total SARA score from baseline after 48 weeks of treatment. In accordance with the predetermined statistical analysis protocol, the change in total SARA score was compared using ANCOVA, with the baseline as the covariate, and the study groups (l-arginine and placebo group) as the fixed-effects groups. In the main analysis, missing values were supplemented using Last Observation Carried Forward (LOCF). As a sensitivity analysis, a Mixed-Effects Model for Repeated Measures (MMRM) analysis was performed, with the change from baseline at weeks 0, 4, 8, 16, 24, 32, 40, and 48 as the response variables. The model included treatment group, time, and baseline score as fixed effects, and subjects as random effects.

The secondary endpoints were 1) SARA walking + standing score, 2) each of the eight SARA scales at 0, 4, 8, 16, 24, 32, 40, and 48 weeks, and 3) TUGT, BDI-II, CGI, PGI-I, and SF-8. For 1) and 2), complete-case ANCOVA, with baseline as the covariate and the l-arginine group as the fixed effect. TUGT, BDI-II, CGI, PGI-I, and SF-8 were also evaluated for changes at 48 weeks and baseline. Based on the predetermined protocol, multiplicity was not considered in the statistical analysis of secondary outcome measures.

**Fig. 3:**
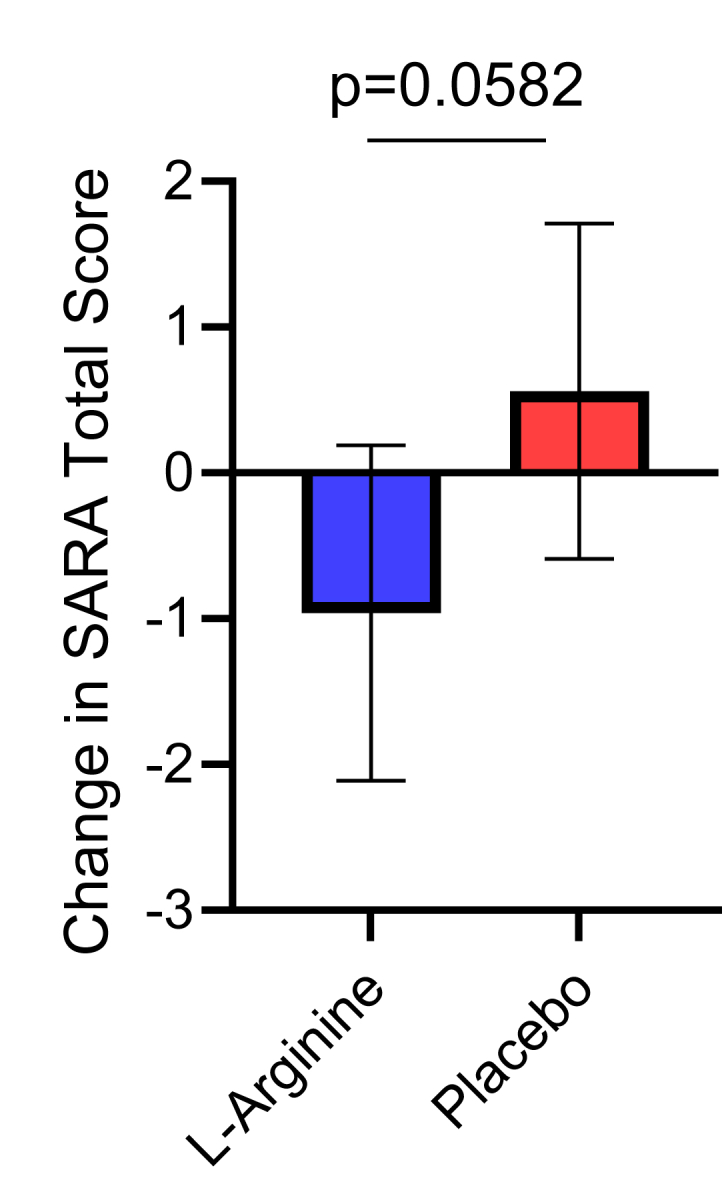
A**djusted least-square means and 95% CIs of changes in total SARA scores from baseline at 48 weeks in the FAS (primary endpoint)**. *P*-values were calculated using the ANCOVA method. ANCOVA, analysis of covariance; FAS, full analysis set; SARA, Scale for the Assessment and Rating of Ataxia.

**Fig. 4:**
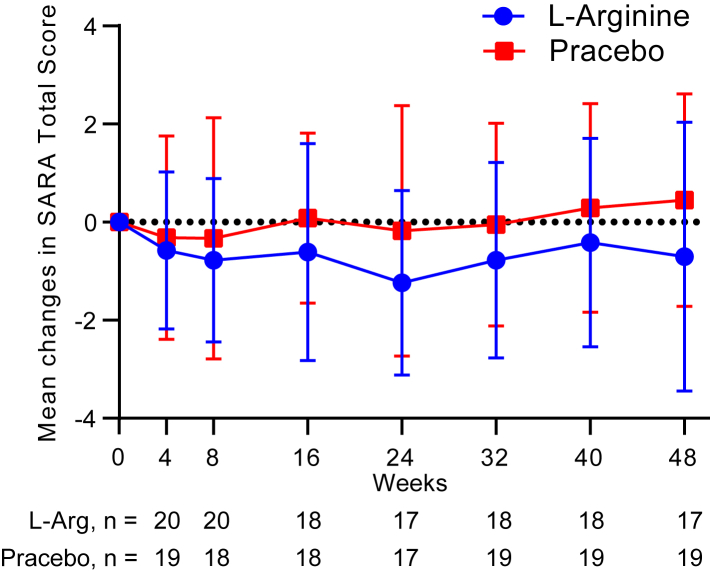
**Changes in total SARA scores from baseline in the FAS (sensitivity analysis)**. Data are shown as the mean ± SD. FAS, full analysis set; SARA, Scale for the Assessment and Rating of Ataxia.

The primary endpoint of “change in SARA total score after 48 weeks of treatment” in the FAS analysis was −0.96 (adjusted least-square [LS] mean; adjusted 95% confidence interval [CI] −2.07 to 0.15) in the l-arginine group, and 0.56 (adjusted LS mean; adjusted 95% CI: −0.55 to 1.67) in the placebo group. The difference between the two groups was −1.52 (95% CI: −3.10 to 0.06, *P =* 0.0582) (Table 3, Fig. 3). In the PPS analysis, the difference between the two groups was −1.13 (95% CI: −2.79 to 0.53, *P =* 0.1754) (Table 3). As a sensitivity analysis, MMRM was analyzed. The change from baseline was greater in the l-arginine group than in the placebo group at all assessment time points (4, 8, 16, 24, 32, 40 and 48w) (Fig. 4). The change in SARA total score after 48 weeks of treatment was −1.13 (adjusted LS mean; adjusted 95% CI: −2.81 to 0.55, *P* = 0.1821). However, MMRM results showed no significant differences between the two groups at any time point (Table 4).

**Table 3:** Primary endpoint: SARA total score change from baseline to 48 weeks.

	L-Arginine			Placebo			P-value
	ｎ	Adjusted LS Mean	Adjusted 95% CI	ｎ	Adjusted LS Mean	Adjusted 95% CI	
FAS	20	−0.96	[−2.07, 0.15]	20	0.56	[−0.55, 1.67]	0.0582
PPS	18	−0.72	[−1.90, 0.47]	19	0.42	[−0.74, 1.57]	0.1754

CI, Confidence interval; FAS, Full analysis set; LS Mean, Least square mean;

PPS, per protocol set; SARA, Scale for the Assessment and Rating of Ataxia;

SE, standard error.

**Table 4:** Sensitivity analysis and Secondary endpoints: Change from baseline.

	L-Arginine					Placebo					*P* value
	ｎ	Mean	SD	Adjusted LS Mean	Adjusted SE	ｎ	Mean	SD	Adjusted LS Mean	Adjusted SE	
Total SARA Score											
4 w	20	−0.58	1.600	−0.51	0.41	19	−0.32	2.076	−0.35	0.42	0.7865
8 w	20	−0.78	1.666	−0.71	0.45	18	−0.33	2.461	−0.28	0.47	0.5158
16 w	18	−0.61	2.213	−0.72	0.45	18	0.08	1.734	0.13	0.45	0.1938
24 w	17	−1.24	1.880	−1.28	0.52	17	−0.18	2.555	−0.05	0.53	0.1073
32 w	18	−0.78	1.994	−0.87	0.48	19	−0.05	2.068	−0.04	0.47	0.2252
40 w	18	−0.42	2.130	−0.47	0.52	19	0.29	2.130	0.31	0.51	0.2906
48 w	17	−0.71	2.745	−0.68	0.59	19	0.45	2.172	0.45	0.57	0.1821
TUGT (sec)											
48 w	17	0.93	4.27	–	–	19	6.60	11.52	–	–	–
BDI-II											
48 w	17	1.1	5.18	–	–	19	2.2	6.95	–	–	–
SF-8											
48 w	17	0.1	3.38	–	–	19	0.5	4.85	–	–	–
CGI-S											
48 w	17	0.0	0.35	–	–	19	0.4	0.61	–	–	–

BDI-II, Beck Depression Inventory-II; CGI-S, Clinical Grobal Impression Severity;

SARA, Scale for the Assessment and Rating of Ataxia; SF-8, Short Form-8; TUGT, Timed Up and Go Test; w, week.

The results of complete-case ANCOVA for the SARA “walking and standing total score” and individual SARA scores are shown in Supplementary Table S7. The TUGT results are shown in Supplementary Table S8. The BDI-II and SF-8 results are shown in Table 4 and Supplementary Table S8, the CGI-S results in Supplementary Tables S8 and S9, the CGI-I results in Supplementary Table S10, and the PGI-I results in Supplementary Table S11. In accordance with the predetermined statistical analysis protocol, multiplicity was not considered in the statistical analysis of the secondary outcome measures.

For CGI-I at 48 weeks, minimally improved 5.9%, no change 76.5% and minimally worse 17.6% in the active drug group. In the placebo group, minimally improved 0%, no change 63.2% and minimally worse 36.8% (Supplementary Figure S10). For PGI-I at 48 weeks, 17.6% were minimally improved, 52.9% no change and 17.6% minimally worse in the active drug group. In the placebo group, minimally improved 5.3%, no change 47.4% and minimally worse 36.8% (Supplementary Figure S11).

The SARA score at 52 weeks, which was 4 weeks after the end of investigational drug administration, was assessed as an additional endpoint not included in the study protocol. The difference in SARA changes from baseline between the l-arginine and placebo groups was −1.22 at 52 weeks, which was similar to that at 48 weeks. ANCOVA showed no significant difference at 52 weeks between the l-arginine and placebo groups (Supplementary Figure S2, Supplementary Table S12).

Finally, we calculated the required sample size to detect a statistically significant difference in a phase 3 study using dosages and endpoints as in this trial. Based on the observed changes in SARA scores between the arginine and placebo groups in this trial, employing a significance level of 5% (two-sided) and a power of 80%, the sample size calculation for comparing the means of the two groups indicated a necessity for 41 participants in each group.

## Discussion

We conducted a Phase 2 trial to evaluate the safety and therapeutic efficacy of l-arginine in SCA6 patients. In the primary endpoint, namely the change in SARA total score after 48 weeks of treatment, the difference between the l-arginine group and the placebo group was −1.52 (95% CI: −3.101 to 0.055, *P* = 0.0582) (Table 3, Fig. 3). The change from baseline was consistently showed negative values in the l-arginine group during the study period (Fig. 4). These results suggest that l-arginine may ameliorate the symptoms of SCA6 patients. Based on the effect size observed in this pilot study, we estimated that a future study would require approximately 41 patients per group to have 80% power to detect a statistically significant difference, assuming the true effect size is equal to what we observed. However, it's important to note that this estimation comes with considerable uncertainty. The true effect size may differ from our observation, and there is no guarantee of a significant result even with this sample size. This estimation should be viewed as a starting point for planning future studies, rather than a definitive requirement.

This phase 2 pilot study was primarily designed to estimate the effect size of l-arginine treatment in SCA6 patients, rather than to demonstrate statistically significant efficacy. We acknowledge the challenge in balancing the pilot nature of the study with the relevance of our findings. While we observed a trend towards improvement in the l-arginine group, it's crucial to note that this study was not powered to detect statistically significant differences. The observed effect size and its confidence interval provide valuable information for planning future, larger-scale studies.

l-arginine has been shown to inhibit conformational changes that occur in the expanded polyglutamine tracts of disease-causing proteins, and to prevent their cytotoxicity.^20^ This experiment was conducted on SCA1 and SBMA model animals, and it was hypothesized that similar effects could be obtained for SCA6, another polyglutamine disease. Based on this, the expected effect of l-arginine was to suppress the progression of neurological symptoms of SCA6 patients.

In this study, SCA6 was selected as the target. This is because SCA6 has a large number of cases in Japan, making it relatively easy to recruit eligible patients, the range of CAG repeats in SCA6 is small, and phenotypic progression is relatively uniform. However, SCA6 progresses more slowly than other spinocerebellar degenerations, and there is a presumption that a large sample size is required to demonstrate a treatment effect.^30^ Our study was designed as an initial step in examining the potential of l-arginine in SCA6 and had a small sample size.

The inclusion criterion of patients with SARA scores of 10 or higher is a key aspect of this trial. Patients in the early stages of disease are excluded from the study, reducing the likelihood of detecting disease-modifying effects in mild cases. However, there have been several reports of placebo effects in mild cases of SCA, which can complicate the interpretation of study results and pose challenges in conducting clinical trials.^23^^,^^24^ Considering these factors, we designed this trial to preliminarily exclude mild cases. Future studies may consider including patients in the earlier stages of the disease to better capture the potential disease-modifying effects of l-arginine in SCA6.

In this study, the daily oral dose was capped at 33 g/day. Since the oral medication was packaged in 3 g increments, the dosage was administered in 3 g increments, corresponding to a certain range of body weights (Supplementary Table S1). Although this upper limit was set to account for the difficulty of ingesting large quantities of granule medicines, dosages based on this setting resulted in a relatively insufficient internal dose for patients weighing more than 70 kg. In the active drug group, 7 out of 20 patients weighed more than 70 kg. This could potentially have affected the study results. Specifically, a higher upper dose limit for oral administration could have resulted in a higher therapeutic effect.

Surprisingly, the l-arginine group showed an improvement in neurological symptoms from baseline, with a SARA change of −0.96 (95% CI: −2.07 to 0.15) at 48 weeks. This improvement may be a result of inhibition of the toxic conformational change and aggregation of the disease-causing proteins, leading to a restoration in the function of cells that were in a state of dysfunction. Suppression of the progression of cellular degeneration may also improve the motor learning effects of the cerebellum.^31^ It is noteworthy that even at four weeks after the end of treatment with the investigational drug, the difference in SARA scores between the groups still remained similar as at the end of treatment. This indicates that the effect of l-arginine is not transient, but rather persists for a certain duration, indicating the disease-modifying benefits of l-arginine treatment. In the future, the extent to which this improvement is sustained should be verified by long-term drug administration. Long-term clinical trials may demonstrate greater improvements.

The mean annual SARA worsening rate in SCA6 patients was reported to range from 0.80 to 0.87.^30^^,^^32^^,^^33^ In a study on Japanese SCA6 patients, the mean annual SARA worsening rate varied depending on the base SARA score, ranging from 1.48 ± 1.86 for patients with a SARA score of 0–24.5, to 0.48 ± 1.42 for patients with a SARA score of 25 or higher.^34^ The SARA 48-week exacerbation rate for the placebo group in this study was 0.56 ± 0.55, which was comparable to or slightly lower than the exacerbation rate estimated from these previous reports. The high placebo effect in patients with neurodegenerative diseases, particularly in those with cerebellar ataxia, has been noted as a confounding factor in clinical trials.^23^ Furthermore, a higher placebo effect was observed in patients with lower SARA scores.^24^ On the basis of these findings, we recruited patients with SARA scores above this threshold. In this trial, we found a −1.52 (95% CI: −3.10 to 0.06, *P =* 0.0582) difference between the l-arginine group and the placebo group, despite the small sample size. This difference might be attributable to the careful patient selection that potentially minimized the placebo effect.

An important outcome of this trial in SCA6, a progressive disease, was the SARA improvement of −0.96 (95% CI: −2.07 to 0.15) in the active drug group and −1.52 (95% CI: −3.10 to 0.06, *P* = 0.0582) compared to the placebo group. However, whether this is a clinically meaningful improvement that patients can perceive needs to be considered from multiple perspectives. First, the Minimal Important Change (MIC) or Minimal Clinically Important Difference (MCID) of SARA in SCA6 should be considered. In a study of multiple types of ataxia diseases, the MIC of SARA was reported to be 0.7,^35^ but there is no adequate study specifically for SCA6. The MCID of the Ataxia Assessment Scale is still insufficiently studied,^36^ and it is hoped that a consensus can be reached.^37^ SARA is also a clinician-reported outcome measure and not a scale to assess patient-perceived improvement.^37^ The PGI-I was used as the scale for assessing patient-perceived improvement in this trial. At the end of the trial, the actual drug group reported being minimally improved in 5.9% of cases and no change in 76.5% of cases, indicating that few patients perceived improvement (Supplementary Figure S11). However, the PGI-I is a seven-point scale of improvement, making it difficult to assess small variations. A more sensitive improvement scale is preferred for assessing efficacy in slowly progressive diseases such as SCA6. One candidate for this is the Friedreich Ataxia Rating Scale (FARS-ADL). It consists of nine items (0–36 points) and has been shown to be useful for assessing ADL in ataxias other than Friedreich Ataxia.^35^ Furthermore, it is often used in international registries of cerebellar ataxia, and data are accumulating. Incorporating the FARS-ADL into the assessment scale in the next phase of clinical trials could enable more precise assessment of improvement.

Regarding the tolerability of l-arginine, the medication adherence rate was 97.2%. Although there were concerns that a high dosage of up to 12 g per dose (Supplementary Table S1) and the powder formulation might decrease the medication adherence rate, it can be concluded that the tolerability was sufficiently high. ARs were more common in the l-arginine group than in the placebo group. Of these, two serious ARs were observed in the l-arginine group: one case (5.0%) of pneumonia (death) and one case (5.0%) of abnormal liver function (recovery) (Table 2).

Blood arginine levels in the active drug group decreased by approximately 15% at 4 and 48 weeks (Fig. 2). Medication adherence rate was high throughout the trial period, and the timing of blood sampling was standardized to allow at least 6 h after administration. This suggests that metabolic changes associated with long-term high-dose oral administration of arginine may have occurred in individual subjects. Further investigation into these results may be enhanced by multiple testing in a larger number of cases in the next phase trial.

This study has several limitations. First, the lack of CAG repeat length data is a significant limitation of this study. In polyglutamine disease patients, it has been reported that CAG repeat length may be associated with SARA deterioration rate.^3^^,^^30^^,^^33^ In future studies, collecting and adjusting for CAG repeat length will be crucial for more accurate assessment of treatment effects and potential stratification of patients. Second, the dose of L-arginine in this study was set at 0.38 g/kg/day, based on the dose approved in Japan for urea cycle disorders. In the nonclinical proof of concept study, mice were administered a 6% l-arginine-HCl solution. The human equivalent dose to that administered to mice based on Food and Drug Administration criteria is 0.80 g/kg/day as l-arginine.^20^^,^^38^ In the future, once data on the safety of long-term oral administration of higher doses of l-arginine are accumulated, the administration of higher doses to SCA6 patients should be considered. Third, our study did not employ stratification by site, and we did not assess potential cluster or center effects. This limitation should be considered when interpreting our results and should be addressed in future, larger studies.

The primary endpoint, change in total SARA score after 48 weeks of treatment, showed a −1.52 (95% CI: −3.10 to 0.06, *P =* 0.0582) difference between the l-arginine group and the placebo group. However, serious adverse drug reactions occurred in the l-arginine group; therefore, safety precautions should be taken. Based on these results, we conclude that a phase 3 study of l-arginine for SCA6 can be planned with a 48-week observation period and the primary endpoint of “change in total SARA score”, as in the present study.

## Contributors

TI, MT, KY, YN, KS, and OO contributed to the conception and design of the study. TI, YT, KI, KI, MH, TY, and YN were investigators who conducted the study and collected the data. TI, YK, YN, and OO directly accessed and verified the data. EM, KY, YN, and OO provided advice regarding the data analysis. The first draft of the manuscript was written by TI, and YN and OO revised the manuscript. YN and OO contributed to funding acquisition. All authors contributed to data interpretation, and to writing, reviewing, and approving the final manuscript for submission.

## Data sharing statement

The data underlying this Article will be shared with researchers on reasonable request to the corresponding author. Data will be available at the time of publication and for a minimum of 5 years from the end of the trial.

## Declaration of interests

TI belongs to an endowment department supported by NSG Holdings Co., Niigata, Japan.

MH has received honoraria from Sumitomo Pharma Co., Ono Pharmaceutical Co., Otsuka Pharmaceutical Co., Novartis International AG, Kyowa-Kirin Co., Eisai Co., and Takeda Pharmaceutical Co.; and has received a research grant from Takeda Pharmaceutical Co.

YN previously belonged to the Department of Neurotherapeutics, Osaka University Graduate School of Medicine, which is an endowment department supported by Nihon Medi-Physics Co., AbbVie GK., Otsuka Pharmaceutical Co., Kyowakai Med. Co., Fujiikai Med. Co., Yukioka Hosp., Osaka Gyoumeikan Hosp., Kyorin Co., and Tokuyukai Med. Co.

YN and ENM have filed a patent application related to this work (PCT/JP2017/023162), which has been granted in Japan (JP6933380B2), Europe (EP3476391B1; validated in Spain as ES2896224T3), and China (CN109310659B), and is pending in the United States of America (US20190314311A1).

OO has received speaker honoraria from Kyowa Hakko Kirin Co., Ltd., Bristol-Myers Squibb, Ono Pharmaceutical Co., Ltd., Mitsubishi Tanabe Pharm, Takeda, Daiichi-Sankyo, FUJIFILM, SANOFI, and FP-pharm. OO have a patent for diagnosis of HTRA1 associated disorders.

## Appendix A Supplementary data

Supplement Figures

Protocol_EN
