# Supplementary material for: L-arginine in patients with spinocerebellar ataxia type 6: a multicentre, randomised, double-blind, placebo-controlled, phase 2 trial
Source: eClinicalMedicine. 2024 Nov 25;78:102952. doi: 10.1016/j.eclinm.2024.102952 (PMC11701440; doi:10.1016/j.eclinm.2024.102952)
Supplement: Appendix File [file mmc1.docx]

**Appendix File1**

**Collaborators**

**Niigata University:** Masato Kanazawa, Akio Yokoseki, Takayoshi Tokutake, Etsuji Saji, Takuya Konno, Akihiro Sugai, Sou Kasahara, Yutaka Otsu, Rei Kato, Yuka Koike, Shoichiro Ando, Tomomi Yamada, Takuma Yamagishi, Shingo Koide, Kae Kobayashi, Mototsugu Tanaka, Seiichi Maruyama, Ryo Nishino, Nobutaka Kitamura, Masahiro Ishizawa, Takahiro Tanaka, Atsushi Hashimoto, Asami Tanaka , Satomi Ikarashi, Ayumi Namekata, Yuko Nakano, Mikako Hirasawa, Ai Shibayama, Hiroka Imamura, Atsuko Nagasawa, Miki Umeda, Junko Sato, Moemi Minagawa, Takeyuki Watanabe, Ai Otaki, Megumi Yokoyama, Kyoko Suganuma, Haruna Miyazawa and Ryohei Terashima

**National Center of Neurology and Psychiatry:** Yuji Saitoh, Shinji Oda, Komei Shimokawa, Naoya Gogu, Yukiko Nakamura, Yuko Mori, Fumiko Kurumada, Naoko Nishimura, and Reiko Saiga

**Osaka University:** Hideki Mochizuki, Toshihide Takeuchi, Yasuyoshi Kimura, Jyunki Jinno, Takashi Miwa, Takahiro Ajiki, Tomoya Wadayama, Tomoko Yamauchi, Tamao Nakamura, Ayumu Nakamura, Makoto Hideshima, Tomoya Chiba, Namie Hasuike, and Noriko Uezono

**Institute of Science Tokyo:** Taro Ishiguro, Nobuo Sanjo, Yoichiro Nishida, Hiroya Kuwahara, Ryuji Koike, Kazuhiko Arakawa, Eri Kato, Shitsuko Shimano, Yuki Hagiwara, Haruko Hiraki, and Shoko Onoda

**Kindai University:** Tomoya Taminato, Yoshiyuki Mitsui, Kazumasa Saigoh, Makoto Samukawa, Yuta Fukumoto, Toshihide Takeuchi, Chiharu Isono, Yasuhiro Kidera, and Yoko Maeda

**Appendix File2**

**Site List**

Coordinating Investigator: Osamu Onodera, Niigata University

Site 1. Niigata University Medical & Dental Hospital, Niigata, Japan

Principle investigator: Tomohiko Ishihara, Niigata University

Number of registered cases: 10

Site 2. NCNP Hospital, Tokyo, Japan

Principle investigator: Yuji Takahashi, National Center of Neurology and Psychiatry

Number of registered cases: 10

Site 3. Institute of Science Tokyo Hospital, Tokyo, Japan

Principle investigator: Takanori Yokota, Institute of Science Tokyo

Number of registered cases: 11 (including 1case of withdrawal before administration)

Site 4. Osaka University Hospital, Suita, Japan

Principle investigator: Kensuke Ikenaka, Osaka University

Number of registered cases: 5

Site 5. Kindai University Hospital, Kindai University, Osaka, Japan

Principle investigator: Makito Hirano

Number of registered cases: 5
