# Supplementary figures and images for: L-arginine in patients with spinocerebellar ataxia type 6: a multicentre, randomised, double-blind, placebo-controlled, phase 2 trial
Source: eClinicalMedicine. 2024 Nov 25;78:102952. doi: 10.1016/j.eclinm.2024.102952 (PMC11701440; doi:10.1016/j.eclinm.2024.102952)

Supplement Figure 1

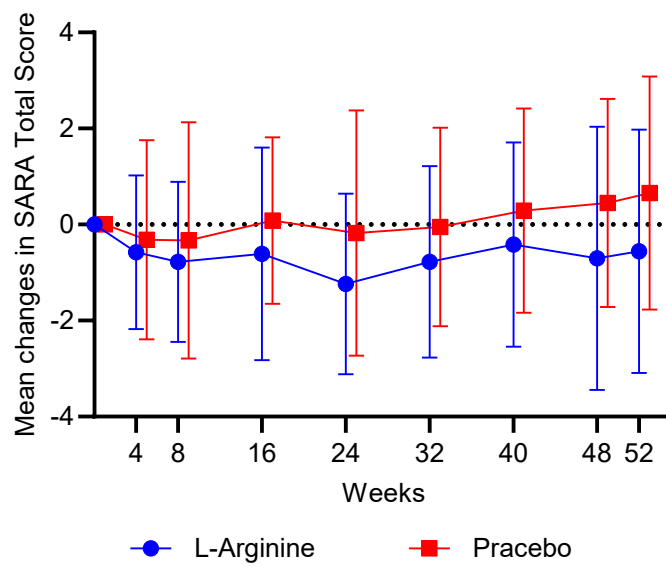

Supplement: Supplement Figures [file mmc3.pdf]
