# Supplementary material for: L-arginine in patients with spinocerebellar ataxia type 6: a multicentre, randomised, double-blind, placebo-controlled, phase 2 trial
Source: eClinicalMedicine. 2024 Nov 25;78:102952. doi: 10.1016/j.eclinm.2024.102952 (PMC11701440; doi:10.1016/j.eclinm.2024.102952)
Supplement: Protocol_EN [file mmc4.docx]

**Protocol**

Title:

A Placebo-Controlled, Double-Blind, Randomized,

Group-Controlled Trial Evaluating the Efficacy and Safety of AJA030

in Spinocerebellar Degeneration.

(Phase II study)

Coordinating Investigator:

Osamu Onodera

Department of Neurology,

Niigata University Medical and Dental Hospital

Clinical protocol number: AJA030-002

Version 3.1

Date of issue; 5 November 2020.

| This protocol contains information that is provided exclusively to investigators, sub-investigators, investigative medical centres and investigative review committees who are willing to participate in this clinical trial.  Please do not disclose or divulge the contents of this protocol to third parties. |
| --- |

# Table of Contents

[Table of contents 1](#_Toc54869059)

[1. Summary of the clinical trial protocol. 4](#_Toc54869060)

[2. Definition of words and abbreviations. 9](#_Toc54869061)

[3. Development history. 10](#_Toc54869062)

[3.1 Target diseases 10](#_Toc54869063)

[3.1.1 Importance of targeted diseases 10](#_Toc54869064)

[3.2 Name and description of the active drug 10](#_Toc54869065)

[3.2.1 Name of the active drug 10](#_Toc54869066)

[3.2.2 Overview of the active drug 10](#_Toc54869067)

[3.2.3 Development history of the AJA030 10](#_Toc54869068)

[3.2.4 Non-clinical results of the AJA030 11](#_Toc54869069)

[3.2.5 L-arginine clinical trial results 13](#_Toc54869070)

[3.2.6 Predicted benefits and disadvantages for subjects 13](#_Toc54869071)

[4. Objectives of the clinical trial. 14](#_Toc54869072)

[4.1 Reasons for deciding that the trial is feasible](#_Toc54869073) 14

[4.2 Significance of this clinical trial 14](#_Toc54869074)

[5. Subject. 14](#_Toc54869075)

[5.1 Inclusion criteria 14](#_Toc54869076)

[5.1.1 Basis for setting inclusion criteria 15](#_Toc54869077)

[5.2 Exclusion criteria. 15](#_Toc54869078)

[5.2.1 Basis for setting exclusion criteria 16](#_Toc54869079)

[6. Explanation and consent to subjects (informed consent). 16](#_Toc54869080)

[6.1 Timing and method of explaining consent and obtaining consent 16](#_Toc54869081)

[6.2 Revision of consent and explanation documents 16](#_Toc54869082)

[6.3 Contents of consent explanatory document 16](#_Toc54869083)

[7. Investigational drugs. 17](#_Toc54869084)

[7.1 Name of investigational drug 17](#_Toc54869085)

[7.2 Composition, properties and method of preparation 17](#_Toc54869086)

[7.2.1 Composition 17](#_Toc54869087)

[7.2.2 Properties 17](#_Toc54869088)

[7.3 Containers, packaging, labelling and storage conditions 17](#_Toc54869089)

[7.4 Storage of investigational medicinal products 19](#_Toc54869090)

[8. Methods of clinical trials 19](#_Toc54869091)

[8.1 Type of clinical trial design 19](#_Toc54869092)

[8.1.1 Rationale for setting up the clinical trial design 19](#_Toc54869093)

[8.2 Target number of cases 19](#_Toc54869094)

[8.2.1 Basis for setting the target number of cases 19](#_Toc54869095)

[8.3 Procedures for conducting clinical trials 19](#_Toc54869096)

[8.3.1 Procedure for obtaining consent and randomising subjects 19](#_Toc54869097)

[8.3.2 Investigational drug administration 20](#_Toc54869098)

[8.3.3 Basis for setting doses 20](#_Toc54869099)

[8.3.4 Rationale for setting durations of administration 23](#_Toc54869100)

[8.4 Assessment of safety and efficacy 23](#_Toc54869101)

[8.4.1 Safety assessment 23](#_Toc54869102)

[8.4.2 Efficacy primary endpoint 23](#_Toc54869103)

[8.4.3 Basis for establishing efficacy primary endpoints 23](#_Toc54869104)

[8.4.4 Efficacy secondary endpoints 23](#_Toc54869105)

[8.5 Observations/test items and trial implementation schedule 24](#_Toc54869106)

[8.5.1 Schedule of clinical trials 24](#_Toc54869107)

[8.5.2 Observations/test items 27](#_Toc54869108)

[9. Management of test subjects. 29](#_Toc54869109)

[9.1 Health care 29](#_Toc54869110)

[9.2 Prohibited drugs and drug-free treatment 29](#_Toc54869111)

[9.3 Follow-up after completion or interruption of a clinical trial (when necessary)](#_Toc54869112) 29

[10. Ensuring the safety of test subjects. 30](#_Toc54869113)

[10.1 Ensuring the safety of subjects 30](#_Toc54869114)

[10.2 Criteria for discontinuation of a clinical trial per subject 30](#_Toc54869115)

[10.3 Procedures for discontinuation of a clinical trial per subject 30](#_Toc54869116)

[11. Adverse events. 31](#_Toc54869117)

[11.1 Definition of adverse events 31](#_Toc54869118)

[11.2 Assessment of adverse events 31](#_Toc54869119)

[11.2.1 Determination of adverse events 31](#_Toc54869120)

[11.2.2 Severity of adverse events 31](#_Toc54869121)

[11.2.3 Causal relationship between investigational drug and adverse events 32](#_Toc54869122)

[11.2.4 Predictability of adverse events 32](#_Toc54869123)

[11.2.5 Severity of adverse events 32](#_Toc54869124)

[11.3 Main adverse events and reactions expected in this trial 32](#_Toc54869125)

[11.3.1 Major adverse events foreseen in this trial 32](#_Toc54869126)

[11.4 Response to adverse events 33](#_Toc54869127)

[11.4.1 Dealing with anticipated adverse events 33](#_Toc54869128)

[11.4.2 Follow-up of adverse events that cannot be ruled out as causally related to the investigational drug 33](#_Toc54869129)

[11.4.3 Recording of adverse events when they occur 33](#_Toc54869130)

[11.5 Handling of serious adverse events 33](#_Toc54869131)

[11.6 Handling of safety information obtained during clinical trials 34](#_Toc54869132)

[11.7 Pregnancy and maternity and when obtaining information on childbirth 34](#_Toc54869133)

[12. Effectiveness and Safety Assessment Committee. 35](#_Toc54869134)

[13. Ethical and scientific conduct of clinical trials. 35](#_Toc54869135)

[13.1 Compliance with laws and regulations 35](#_Toc54869136)

[13.2 Clinical trial review committees 35](#_Toc54869137)

[13.3 Preservation of confidentiality of subjects 35](#_Toc54869138)

[14. Statistical matters. 35](#_Toc54869139)

[14.1 Definition of analysis population 35](#_Toc54869140)

[14.1.1 Population subjected to safety analysis 35](#_Toc54869141)

[14.1.2 Efficacy analysis population 36](#_Toc54869142)

[14.2 Criteria for handling cases 36](#_Toc54869143)

[14.3 Analysis items/methods 36](#_Toc54869144)

[14.3.1 Subject background 36](#_Toc54869145)

[14.3.2 Safety analysis 36](#_Toc54869146)

[14.3.3 Analysis of efficacy endpoints 37](#_Toc54869147)

[14.3.4 Analysis of plasma drug concentrations 38](#_Toc54869148)

[14.4 Interim analysis 38](#_Toc54869149)

[14.5 Procedures for preparing and modifying statistical analysis plans 38](#_Toc54869150)

[15. Deviations from the study protocol, amendments to the study protocol, suspension or discontinuation of the entire study. 38](#_Toc54869151)

[15.1 Compliance with the clinical trial protocol 38](#_Toc54869152)

[15.2 Deviations from the clinical trial protocol 38](#_Toc54869153)

[15.3 Revision of the clinical trial protocol 38](#_Toc54869154)

[15.4 Suspension or discontinuation of the entire clinical trial 39](#_Toc54869155)

[15.4.1 Criteria for suspending or discontinuing the overall clinical trial 39](#_Toc54869156)

[15.4.2 Procedures for suspending or discontinuing an entire clinical trial 39](#_Toc54869157)

[16. Case report. 39](#_Toc54869158)

[17. Preservation of documents or records. 39](#_Toc54869159)

[17.1 Documents or records to be kept and where they are kept 39](#_Toc54869160)

[17.2 Duration of storage 40](#_Toc54869161)

[18. Quality control and quality assurance of clinical trials. 41](#_Toc54869162)

[18.1 Quality control 41](#_Toc54869163)

[18.1.1 Monitoring 41](#_Toc54869164)

[18.2 Quality assurance 41](#_Toc54869165)

[18.2.1 Data management 41](#_Toc54869166)

[18.2.2 Audit 41](#_Toc54869167)

[19. Direct access to original documents and other materials. 41](#_Toc54869168)

[20. Clinical trial costs and compensation for damage to health. 41](#_Toc54869169)

[20.1 Sources of funding and conflicts of interest in this clinical trial 41](#_Toc54869170)

[20.2 Subject cost-sharing related to clinical trials 42](#_Toc54869171)

[20.3 Compensation for health damage 42](#_Toc54869172)

[21. Arrangements for attribution of results and publication of trial results. 42](#_Toc54869173)

[22. Duration of the clinical trial. 42](#_Toc54869174)

[23. Clinical trial implementation system. 42](#_Toc54869175)

[24. References. 43](#_Toc54869176)

**Annex 1: Clinical trial implementation system**

# Summary of the clinical trial protocol

| **Trial subject** | A placebo-controlled, double-blind, randomised, group-based study evaluating the efficacy and safety of AJA030 in spinocerebellar degeneration (Phase II). |
| --- | --- |
| **Objectives of the clinical trial** | To assess the efficacy and safety of AJA030 in spinocerebellar degeneration. |
| **Clinical Trial Design** | Placebo-controlled, double-blind, randomised, group-controlled study. |
| **Target disease** | Spinocerebellar degeneration |
| **Inclusion**  **criteria** | 1. Patients with genetically diagnosed autosomal dominant hereditary spinocerebellar degeneration (SCA6). Note 1) 2. Patients aged 20 years or older at the time of informed consent. 3. Patients who scored 1 or more In SARA(Scale for the Assessment and Rating of Ataxia) gait scores in the pre-enrollment test and had a SARA "sum" score of 10 or more. 4. Patients who are able to walk 10m or more with or without assistive devices in the pre-enrollment test. 5. Patients for whom written informed consent is obtained from the individual for participation in the clinical trial.   Note 1) Genetic diagnosis of the patient is not indispensable, when the disease type is confirmed by the genetic diagnosis in the family. |
| **Exclusion criteria** | 1) Patients with gait or balance disorders due to medical conditions other than spinocerebellar degeneration (stroke, brain tumor, head injury, multiple sclerosis, hypothyroidism, alcoholic ataxia, joint disease, others).  2) Patients with arginase deficiency (which exacerbates argininemia), patients with lysinuric protein intolerance who have a greater degree of inhibition of arginine absorption (which causes diarrhea with the use of arginine preparations).  3) Patients with dementia (MMSE<=23), mental illness (psychosis, manic-depressive illness, depression without treatment [Beck Depression Inventory score>=21]), or a history of suicidal attempt.  4) Patients with complications that are considered inappropriate for participation in the study, such as serious heart disease, liver disease, kidney disease, or hematologic disease.  5) Pregnant or possibly pregnant patients and breastfeeding patients.  6) Patients who are unable to consent to use effective contraceptive methods during the study participation period.  7) Patients who took oral preparations that were judged by the principal investigator or the subinvestigator to be supplements containing algin U or L-arginine as the main ingredient within 30 days prior to the date of informed consent.  8) Patients who participated in another clinical trial within 6 months before the date of obtaining informed consent.  9) Patients who are considered inappropriate to participate in the study by the principal investigator or the subinvestigator. |
| **Investigational drug** | Investigational drug code: AJA030  Active drug: L-arginine (ARGI-U^®^ Combination granules)  Placebo: Preparations that do not contain L-arginine and are visually indistinguishable from the active drug. |
| **Dose** | Active drug group: 0.5 g/kg/day as granules (0.38 g/kg/day as AJA030)  Placebo group: 0.5 g/kg/day as granules (0.0 g/kg/day as AJA030) |
| **Method of administration** | Take a daily dose for three minutes. |
| **Period of administration** | 48 weeks (52 weeks observation period) |
| **Drugs prohibited for use in combination with other drugs** | All concomitant medications (including Seresist^🄬^ (tartirelin hydrate) and Hyltonin Injection^🄬^ (protirelin tartrate hydrate)) are permitted during the study period. However, the dose should be constant for 4 weeks prior to the start of investigational drug administration. Celist^🄬^ (Tartirelin hydrate), Hyltonin Injection^🄬^ (Protilerin tartrate hydrate) and phosphodiesterase 5 inhibitors are not permitted to be started or discontinued during a clinical trial and any cases of initiation or discontinuation will be treated as drop-out or discontinuation cases. |
| **Safety assessment.** | Vital signs (temperature, blood pressure, pulse), body weight, adverse events, haematology, blood biochemistry and urinalysis are performed before enrolment, on the first day of treatment and after 4, 8, 16, 24, 32, 40, 48 and 52 weeks of treatment to assess safety. |
| **Efficacy primary endpoint** | SARA 'total' score after 48 weeks  (The primary endpoint is the change from baseline in the SARA 'total' score at the final assessment of the treatment period.) |
| **Efficacy secondary endpoints** | 1. SARA 'total' scores after 4, 8, 16, 24, 32, 40 and 48 weeks 2. SARA 'Walking, standing' score. 3. SARA Scores for each item (except walking and standing scores) 4. Gravity body sway test (total trajectory length for 30 s, rectangular area) 5. Timed Up and Go Test (TUG) (minimum time) 6. Beck Depression Inventory-II (BDI-II) 7. Clinical Global Impression (CGI) 8. Patient Global Impression of Improvement (PGI-I) 9. Short-Form 8 (SF-8) |
| **Criteria for discontinuation of a clinical trial per subject.** | 1) If the subject withdraws consent to participate in the clinical trial.  2) When it is judged difficult to continue administration of the investigational drug to the subject concerned due to adverse events, etc.  one-tenth of the way from the base to the summit of a mountain  3) Treatment that is judged to have a significant impact on the outcome of the trial, such as the administration of surgical treatment during the trial period.  4) If a female subject becomes pregnant during the study period (if the male subject's partner becomes pregnant, the study can continue).  5) If it is found after registration that the inclusion/exclusion criteria are violated.  6) If the subject is unable to continue the clinical trial for non-medical reasons (e.g. hospital transfer, relocation, etc.)  7) Other cases where the investigator or sub-investigator decides that it is difficult to continue the clinical trial. |
| **Target number of cases** | Total 40 cases (20 in the active drug group and 20 in the placebo group) |
| **Implementing medical institution** | Niigata University Medical and Dental Hospital  Osaka University Hospital  Tokyo Medical and Dental University Hospital  National Center Hospital, National Center of Neurology and Psychiatry  Kindai University Hospital |
| **Duration of the clinical trial and case registration period** | Clinical trial duration: 09/09/2020 - 31/03/2023  Case registration period: 09.09.2020 - 30.09.2021 |

Figure 1: Clinical trial flow

Informed concent

Screening

4 weeks or less

Target number of cases

Registration and allocation

40 cases.

48 weeks.

Taken 3

times/day

Start of investigational drug administration

End of investigational drug administration

Observation and evaluation

1) Safety assessment.

Vital signs, body weight, adverse events, haematological tests, blood biochemical tests and urinalysis before enrolment, at the start of treatment, 4, 8, 16, 24, 32, 40, 48 and 52 weeks after treatment.

2) Efficacy Main assessment.

Change from baseline in SARA 'total score' after 48 weeks of treatment

3) Efficacy secondary evaluation

(i) SARA 'total' scores after 4, 8, 16, 24, 32, 40 and 48 weeks.

(ii) SARA 'Walking, standing' score.

(iii) SARA Scores for each item (except walking and standing scores)

(iv) Gravity body sway test (total trajectory length for 30 s, rectangular area)

(v) Timed Up and Go Test (TUG) (minimum time)

(vi) Beck Depression Inventory-II (BDI-II).

(vii) Clinical Global Impression (CGI).

(viii) Patient Global Impression of Improvement (PGI-I).

(ix) Short-Form 8 (SF-8).

End of clinical trial

Statistical analysis

Summary report preparation

| **Obserrvations** | **Informed Consent** | **Screening** | **Registration/**  **Assignment** | **Duration of investigational drug administration^#6^** | | | | | | | | **Post-observation period** | **Time of discontinuance** |
| --- | --- | --- | --- | --- | --- | --- | --- | --- | --- | --- | --- | --- | --- |
|  |  |  |  | 0th day | 4 weeks | 8 weeks | 16 weeks | 24 weeks | 32 weeks | 40 weeks | 48 weeks | 52 weeks |  |
| Scheduling window | Within 4 weeks prior to registration | | 7 days prior to registration to before  the start of administration | Start Day | ±5 days | ±7 days | ±7 days | ±7 days | ±7 days | ±7 days | ±7 days | ±7 days | - |
| Obtaining consent | ○ |  |  |  |  |  |  |  |  |  |  |  |  |
| Eligibility check | ○ |  | ○ |  |  |  |  |  |  |  |  |  |  |
| Registration and allocation |  |  | ○ |  |  |  |  |  |  |  |  |  |  |
| Administration of an investigational drug |  |  |  |  |  |  |  |  |  |  |  |  | (Check status of internal medication). |
| Subject background |  | ○ |  |  |  |  |  |  |  |  |  |  |  |
| Height |  | ○ |  |  |  |  |  |  |  |  |  |  |  |
| Weight |  | ○ |  | ○^#2^ |  |  |  |  |  |  | ○ | ○ | ○ |
| Vital signs |  | ○ |  | ○^#2^ | ○ | ○ | ○ | ○ | ○ | ○ | ○ | ○ | ○ |
| Distribution of medication logbook |  |  |  | ○ | ○ | ○ | ○ | ○ | ○ | ○ |  |  |  |
| Collection and verification of medication logbook |  |  |  |  | ○ | ○ | ○ | ○ | ○ | ○ | ○ |  | ○ |
| Hematology |  | ○ |  | ○^#2^ | ○ | ○ | ○ | ○ | ○ | ○ | ○ | ○ | <○> |
| Biochemistry |  | ○ |  | ○^#2^ | ○ | ○ | ○ | ○ | ○ | ○ | ○ | ○ | <○> |
| Urinalysis |  | ○ |  | ○^#2^ | ○ | ○ | ○ | ○ | ○ | ○ | ○ | ○ | <○> |
| Pregnancy Test #1 |  | ○ |  | ○^#2^ |  |  |  |  |  |  |  |  |  |
| SARA |  | ○ |  | ○^#2^ | ○ | ○ | ○ | ○ | ○ | ○ | ○ | ○ | <○> |
| Gravity body Sway Test ^#3^ |  | ○ |  | ○^#2^ |  |  |  |  |  |  | ○ | ○ |  |
| TUG |  | ○ |  | ○^#2^ |  |  |  |  |  |  | ○ | ○ | <○> |
| BDI-II |  | ○ |  | ○^#2^ |  |  |  |  |  |  | ○ | ○ | <○> |
| CGI-S |  | ○ |  | ○^#2^ | ○ | ○ | ○ | ○ | ○ | ○ | ○ | ○ | <○> |
| CGI-I |  |  |  |  | ○ | ○ | ○ | ○ | ○ | ○ | ○ | ○ | <○> |
| PGI-I |  |  |  |  | ○ | ○ | ○ | ○ | ○ | ○ | ○ | ○ | <○> |
| SF-8 |  | ○ |  | ○^#2^ |  |  |  |  |  |  | ○ | ○ | <○> |
| MMSE |  | ○ |  | ○^#2^ |  |  |  |  |  |  | ○ |  | <○> |
| C-SSRS |  | 〇 |  |  |  |  |  |  |  |  |  | 〇 | <○> |
| AJA030 PK blood sampling ^#4^ |  |  |  | ○ | ○ |  |  | 〇 |  |  | ○ | ○ | <○> |
| Identification of adverse events^#5^ |  |  |  |  |  |  |  |  |  |  |  |  |  |
| Concomitant medications and therapies |  |  |  |  |  |  |  |  |  |  |  |  |  |

#1: Performed in female subjects of childbearing potential (if pregnancy test is performed with blood, draw approximately 2 mL more blood separately).

#2: If data from pre-registration testing is within one week of the start date of administration, the data may be adopted.

#3: Only cases from Niigata University Medical and Dental Hospital should be conducted.

#4: At the visit to collect the blood level measurement sample, the subject must be instructed to allow at least 6 hours between taking the investigational drug and collecting the sample. At the morning visit, do not take study medication before or after breakfast. At the afternoon visit, do not take the investigational drug before or after lunch. Any investigational medicines skipped for blood concentration measurement should be submitted to the investigator or collaborator as leftover medicines at the time of the visit. There is no need to take them together at the next oral dose.

#5: Adverse event assessments at the start date of dosing are carried out after the administration of the investigational drug.

#6: Weeks 12, 20, 28, 36 and 44 will be telephoned by the site's clinical trial staff to check status and to confirm internal medication status. Tolerance is ±7 days.

<○>: If the study is discontinued during the investigational drug administration period, this should be carried out as far as possibl

# Definition of words and abbreviations

Table 2 Definition of words and abbreviations

| **abbreviation** | **Unabbreviated expression (description).** |
| --- | --- |
| ALT (GPT) | L-Alanine Aminotransferase [Glutamic Pyruvic Transaminase]. |
| AST (GOT) | Asparate Aminotransferase [Glutamic Oxaloacetic Transaminase]. |
| BDI-II. | Beck Depression Inventory-II |
| BUN | Blood Urea Nitrogen |
| CGI | Clinical Global Impression |
| CRP | C-Reactive Protein. |
| C-SSRS | Columbia-Suicide Severity Rating Scale |
| GCP | Good Clinical Practice. |
| GMP | Good Manufacturing Practice |
| γ GTP | gamma-glutamyl transpeptidase |
| LDH | lactate dehydrogenase |
| MMSE | Mini-mental State Examination |
| PGI | Patient Global Impression |
| PK | Pharmacokinetic |
| SARA | Scale for the assessment and rating of ataxia |
| SF-8 | Short-Form 8 |
| SCD | Spinocerebellar Degeneration. |
| TUG | Timed Up and Go Test |
| QOL | Quality of life |

# Development history

## Target disease

Spinocerebellar degeneration

### Importance of target diseases.

Spinocerebellar degeneration (SCD) is a neurodegenerative disease with slow progressive ataxia as its core symptom, affecting an estimated 34 000 people in Japan (1). It is a group of diseases encompassing a diverse range of clinical genetics, with approximately 70% of cases being solitary, mainly multiple system atrophy, and the remaining 30% being hereditary. Among the hereditary forms, polyglutamine diseases, including Machado-Joseph disease/hereditary spinocerebellar ataxia type 3 (MJD/SCA3) and hereditary spinocerebellar ataxia type 6 (SCA type 6: SCA6), account for the majority (1) .

Polyglutamine diseases are inherited neurodegenerative disorders caused by abnormal elongation of the CAG repeat sequence encoding the glutamine chain within the causative gene, and nine disorders are known, including SCA6. In this disease, mutant proteins with abnormally elongated polyglutamine chains acquire high aggregation properties and cause neuronal dysfunction and ultimately cell death. Misfolding and aggregation of the mutant proteins are thought to be the core early molecular pathogenesis, with soluble polymers engulfing normal proteins and causing intermediate pathologies such as impaired transcription, impaired axonal transport, mitochondrial dysfunction, synaptic dysfunction and DNA repair.

A number of treatment intervention trials have been attempted for patients with SCD, most recently idebenone for Friedreich's ataxia (FA) (2) and rehabilitation for multiple cerebellar ataxias (3,4) (4), the effect of riluzole on cerebellar ataxia (5) (6), effect of the smoking cessation drug varenicline on MJD/SCA3 (6) and others were reported. However, all of these effects were temporary and have not resulted in sufficient improvement of symptoms. There are still no effective therapies to control the condition in accordance with the molecular pathomechanism, and the development of new therapies is eagerly awaited.

## Name and description of the active drug

### Name of the active drug drug

AJA030 (ARGI-U^®^ Combination Granules)

### Overview of the active drug

AJA030, the active drug in this study, is a granule preparation containing 605 mg of L-arginine hydrochloride and 500 mg of L-arginine in 1.3 g (total 1000 mg of L-arginine).

　L-arginine is one of the substrates that make up the urea circuit (the ornithine circuit that synthesises urea from ammonia) and is already listed and marketed as a drug for the treatment of congenital urea cycle disorders (7). It has also shown a certain therapeutic effect on the MELAS (mitochondrial encephalomyopathy, encephalopathy, lactic acidosis and stroke-like episodes) form of mitochondrial encephalomyopathy and is already in clinical use.

### Development history of the AJA030

With the aim of overcoming spinocerebellar degeneration, Onodera and his colleagues have long been involved in research relating to the pathogenesis of polyglutamine disease and the development of treatment methods (8). In particular, they have focused on the development of drugs targeting the inhibition of soluble polymerisation, which is considered to be highly cytotoxic; Nagai et al. investigated the aggregation-inhibitory activity of purified polyglutamine proteins using an in vitro assay (US patent) on about 20 chemical chaperones with protein structure stabilising activity. They found that L-arginine markedly inhibited the aggregation of polyglutamine proteins. In cultured cells expressing polyglutamine protein, the aggregation of polyglutamine protein was also inhibited by the addition of L-arginine to the culture medium. Furthermore, when L-arginine was orally administered to the polyglutamine disease model Drosophila, polyglutamine protein aggregation was inhibited and neurodegeneration was suppressed (9). Arginine administration also improved life-span shortening in the nematode model of polyglutamine disease (Tokunaga J et al. unpublished data).

　In a subsequent study, to test the potential of L-arginine as a therapeutic agent for polyglutamine disease, and also for neurodegenerative diseases caused by protein misfolding/aggregation, the efficacy of L-arginine in mouse models of polyglutamine disease was examined. Oral administration of L-arginine hydrochloride (6%) to two models of polyglutamine disease, knock-in mice with spinocerebellar ataxia type 1 (SCA1-KI) and transgenic mice with spherospinal muscular atrophy (SBMA-Tg), significantly reduced motor deficits in SCA1-KI, ubiquitin-positive polyglutamine inclusion body formation in the cortex and hippocampus, significantly reduced spontaneous locomotor activity in SBMA-Tg and significantly increased the number of rises in the cage (Popiel HA et al. unpublished data). Furthermore, oral administration of L-arginine hydrochloride (2%) to SCA1-KI from the onset of locomotor disturbance significantly suppressed locomotor disturbance. These results indicate that L-arginine is expected to inhibit the aggregation of abnormal polyglutamine proteins and suppress neuronal dysfunction, regardless of the gene type.

　Based on the therapeutic efficacy of L-arginine for polyglutamine disease in the basic research described above and its safety in conventional clinical use, a Phase II investigator-initiated clinical trial was planned to provide a safe and effective treatment for spinocerebellar degeneration (limited to polyglutamine disease).

### Non-clinical results of the AJA030.

1) Pharmacological studies on L-arginine

Oral administration of L-arginine hydrochloride (6%) to two different models of polyglutamine disease, knock-in mice with spinocerebellar ataxia type 1 (SCA1-KI) and transgenic mice with spinocerebellar muscular atrophy (SBMA-Tg), significantly reduced motor deficits in SCA1-KI, inhibited ubiquitin-positive polyglutamine inclusion body formation in the cortex and hippocampus, and also significantly suppressed the decrease in spontaneous locomotor activity in SBMA-Tg and significantly increased the number of times the animals stood up in the cage (9). Furthermore, oral administration of L-arginine hydrochloride (2%) to SCA1-KI from the onset of locomotor disturbance significantly suppressed it. These results indicate that L-arginine inhibits the aggregation of abnormal polyglutamine proteins and is expected to have an inhibitory effect on neuronal dysfunction.

2) Safety studies on L-arginine

(1) Safety pharmacology studies

Safety pharmacological effects of arginine evaluated from the literature (10-20) .

In addition, when the effects on blood pressure were tested, no changes in blood pressure or heart rate were observed when 1 g/kg of an equimolar mixture of L-arginine hydrochloride and L-arginine was administered orally to awake, healthy rats (21).

(ii) Single-dose toxicity studies (oral and intravenous)

・Oral administration test

The LD50 value of L-arginine hydrochloride in rats was as high as 12400 mg/kg, and no serious symptoms were observed in both 4-day-old and 6-week-old rats at a dose of 2000 mg/kg of an equimolar mixture of L-arginine hydrochloride and L-arginine, and a rough lethal dose was determined to be 2000 mg/kg or more (22-24).

・Intravenous drug trial

The LD50 value for L-arginine hydrochloride in rats was as high as 3336 mg/kg (2759 mg/kg as L-arginine) (25) . No deaths have been observed in dogs at a dose of 1440 mg/kg of L-arginine (26) .

(iii) Repeated dose toxicity study

・Oral administration test

In a 5-week repeated oral administration study in rats of an equimolar mixture of L-arginine hydrochloride and L-arginine, no changes suggestive of toxicity were observed at 2000 mg/kg/day (1810 mg/kg/day as L-arginine) and the non-toxic dose was determined to be 2000 mg/kg/day (27) .

・Intravenous (IV) drug trial

The non-toxic dose of L-arginine hydrochloride in a 2-week study in rats was 827 mg/kg/day as L-arginine (25) .

(iv) Reproductive and developmental toxicity tests

Effects on reproduction

When rats were fed approximately 2000 mg/kg/day of L-arginine during the period including the period of organogenesis, no effects on maternal or foetal development were observed and no teratogenic effects were observed. In a rat reproduction study using subcutaneous administration of L-arginine, no effects on F0 animals, mating, pregnancy or foetus were observed at a dose of 724 mg/kg/day (28,29) .

### L-arginine clinical trial results.

1) Clinical trials in spinocerebellar degeneration

There have been no clinical trials of L-arginine in spinocerebellar degeneration.

2) Adverse effects in clinical trials and post-marketing surveillance for hyperammonaemia based on congenital urea cycle disorders and lysinuric protein intolerance.

In clinical trials conducted up to the time of approval, adverse reactions were observed in 5 (12.5%) 9 cases out of a total of 40 patients. The adverse reactions included increased AST (GOT) in 2 cases (5.0%), increased ALT (GPT) in 2 cases (5.0%), vomiting in 2 cases (5.0%), nausea/nausea in 1 case (2.5%), itching in 1 case (2.5%) and sleepiness in 1 case (2.5%). In the post-marketing surveillance, adverse reactions (including abnormal laboratory values) were observed in 24 cases (18 cases, 8.1%) out of 222 cases, the main adverse reactions being abnormal liver function in 4 cases (1.8%) and diarrhoea in 4 cases (1.8%) (at the time of the re-examination results).

### Predicted benefits and disadvantages for subjects

1) Anticipated benefits

To date, the efficacy of AJA030 for spinocerebellar degeneration has not been confirmed.

　However, AJA030 is expected to be effective in reducing disease progression by deterring the pathogenesis of spinocerebellar degeneration.

　On the other hand, subjects randomised to the placebo group do not benefit from the trial, such as reducing disease progression.

2) Anticipated disadvantages

Administration of AJA030 may cause undesirable events such as liver function abnormalities, diarrhoea, vomiting, nausea/vomiting, pruritus and drowsiness. Other unanticipated adverse events may also occur, which may require hospital admission or hospital treatment.

# Objectives of the clinical trial

To assess the efficacy and safety of AJA030 in spinocerebellar degeneration.

## Reasons for deciding that the trial is feasible

Spinocerebellar degeneration (SCD) is a neurodegenerative disease whose core symptom is slowly progressive ataxia. It can be solitary or inherited, but in both cases it is mostly caused by abnormal protein aggregation and accumulation. Most hereditary SCD results in abnormal aggregation of polyglutamine proteins and some solitary SCD results in abnormal aggregation of α-synuclein. The conformational changes that lead to abnormal aggregation of this protein are the mainstay of the condition, and suppressing them is considered to be an effective treatment to control the condition. L-arginine, a known chemical chaperone, was found to markedly inhibit the aggregation of polyglutamine protein by Nagai et al. and α-synuclein by Ghosh et al. (30) This suggested that L-arginine, as a chemical chaperone, could be a therapeutic agent for spinocerebellar degeneration associated with aggregation and accumulation of abnormal proteins, regardless of the type of protein.

The active drug AJA030 (L-arginine) is an amino acid preparation already listed in the NHI price list for the treatment of congenital urea cycle disorders as ARGI-U^®^ combination granules. The dose of 0.5 g/kg/day in this study is within the approved dosage and administration, and based on the results of non-clinical studies obtained to date, it was judged that there are no major safety concerns.

　Based on these factors, it was judged feasible to conduct this clinical trial in patientswith spinocerebellar degeneration.

## Significance of this clinical trial.

SCD is a disease that significantly impairs the quality of life (QOL) of patients, and although many therapeutic intervention trials have been attempted, there is still no effective treatment that suppresses the progression of the disease based on the molecular pathomechanism, and the development of new treatment methods is much needed. We decided to conduct this investigator-initiated clinical trial with the aim of providing an effective treatment for SCD.

# Subject.

## Inclusion criteria

Patients with spinocerebellar degeneration who meet all of the following criteria.

1. Patients with genetically diagnosed autosomal dominant hereditary spinocerebellar degeneration (SCA6). Note 1)
2. Patients aged 20 years or older at the time of informed consent.
3. Patients who scored 1 or more In SARA (Scale for the Assessment and Rating of Ataxia) gait scores in the pre-enrollment test and had a SARA "sum" score of 10 or more.
4. Patients who are able to walk 10m or more with or without assistive devices in the pre-enrollment test.
5. Patients for whom written informed consent is obtained from the individual for participation in the clinical trial.

Note 1) Genetic diagnosis of the patient is not indispensable, when the disease type is confirmed by the genetic diagnosis in the family.

### Basis for setting inclusion criteria

1. SCD has a variety of disease types, and each disease type has its own diversity of symptoms. Even within the same disease, there is a great diversity of symptoms and progression. To explore the potential of this drug in a small number of patients in this phase II study, it is desirable to compare the drug in a population that is as homogeneous as possible. However, solitary SCD has a high diversity of symptoms. In polyglutamine disease, which accounts for the majority of hereditary SCD, CAG repeat length has a significant impact on symptoms and severity. Therefore, comparisons are difficult unless CAG repeat lengths are aligned. However, such ideal cases are difficult to incorporate. Among these difficult conditions, one polyglutamine disease, SCA6, is relatively homogeneous in its clinical presentation and progression: in SCA6, the repeat sequence lengths in the affected individuals fall within a certain range and are less diverse. Therefore, the heterogeneity of the clinical picture can be reduced as much as possible. In addition, the frequency of SCA6 is high in Japan and the number of cases is large enough for a clinical trial. For these reasons, the study was limited to SCA6. In addition, SCA31, which has similar symptoms to SCA6 and does not involve polyglutamine aggregates, exists in Japanese SCD. For this reason, the study was limited to cases with a genetic diagnosis of SCA6 in the patient or in the family.
2. The patient must be at least 20 years of age with the capacity to consent.
3. One of the problems in clinical trials for neurological diseases is the sham drug effect. Generally, sham drug effects are more likely to occur in younger patients with milder symptoms. In this study, to eliminate this effect as much as possible, a group with somewhat well-defined symptoms will be included. Therefore, SARA (the Scale for the Assessment and Rating of Ataxia) 'gait' scores of at least 1 point and 'total' scores of at least 10 points were included.
4. In order to carry out the clinical trial and evaluate its efficacy, we considered patients who were able to walk more than 10 m with or without assistive devices, as we considered it difficult to evaluate patients with severe illness who had difficulty walking on their own.

## Exclusion criteria

Patients who fall into any of the following categories should be excluded from coverage.

1) Patients with gait or balance disorders due to medical conditions other than spinocerebellar degeneration (stroke, brain tumor, head injury, multiple sclerosis, hypothyroidism, alcoholic ataxia, joint disease, others).

2) Patients with arginase deficiency (which exacerbates argininemia), patients with lysinuric protein intolerance who have a greater degree of inhibition of arginine absorption (which causes diarrhea with the use of arginine preparations).

3) Patients with dementia (MMSE<=23), mental illness (psychosis, manic-depressive illness, depression without treatment [Beck Depression Inventory score>=21]), or a history of suicidal attempt.

4) Patients with complications that are considered inappropriate for participation in the study, such as serious heart disease, liver disease, kidney disease, or hematologic disease.

5) Pregnant or possibly pregnant patients and breastfeeding patients.

6) Patients who are unable to consent to use effective contraceptive methods during the study participation period.

7) Patients who took oral preparations that were judged by the principal investigator or the subinvestigator to be supplements containing algin U or L-arginine as the main ingredient within 30 days prior to the date of informed consent.

8) Patients who participated in another clinical trial within 6 months before the date of obtaining informed consent.

9) Patients who are considered inappropriate to participate in the study by the principal investigator or the subinvestigator.

### Basis for setting exclusion criteria.

1), 7), 8) To eliminate any influence on the assessment of efficacy or safety.

2), 3), 4) To ensure the safety of subjects and to eliminate any influence on the assessment of safety.

5), 6) To ensure the safety of the subjects and their foetuses and newborns.

9) To avoid incorporating persons who are not suitable for this clinical trial.

# Explanation and consent to subjects (informed consent)

## Timing and method of explaining consent and obtaining consent

The investigators and sub-investigators should carefully consider the appropriateness of asking the subject to participate in the trial, giving due consideration to the subject's health condition, symptoms, age, gender, ability to consent, etc. from the perspective of protecting the subject's human rights.

The investigator or sub-investigator provides sufficient explanation to the subject using the consent explanatory document and obtains the subject's free and voluntary consent in writing after confirming that the subject has fully understood the content. Consent does not have to be given on the day of the explanation.

The consent form should be signed and dated by the subject him/herself. If a trial co-operator provides supplementary explanations, the said co-operator should also sign and date the consent form.

The investigator or sub-investigator keeps the original consent form and gives the subject a copy of the set of documents used to explain consent and a copy of the consent form. The investigator or sub-investigator enters the date the consent was obtained on the case report form.

## Revision of consent and explanation documents

If new findings are obtained after the start of a clinical trial that may affect subject consent, the investigator should promptly revise the consent explanatory document based on the findings. If the investigator revises the consent explanation document, he/she submits the revised document to the head of the investigational medical institution and obtains approval from the Clinical Trial Review Committee. After approval by the Clinical Trial Review Committee, the investigator explains again using the revised consent explanation document and obtains free will consent in writing from the subject for continued participation in the clinical trial.

## Contents of consent explanatory documen

The explanations given to the subjects are as follows.

1. That the clinical trial involves research.
2. Objectives of the study.
3. Methods of this study.
4. Expected duration of the subject's participation in the trial.
5. Number of subjects expected to participate in the clinical trial.
6. Anticipated clinical benefits or disadvantages.
7. Compensation and treatment to which subjects are entitled in the event of health problems related to

the trial.

1. Participation in this clinical trial is of the subject's own free will, and the subject may refuse or

withdraw from participation in this clinical trial at any time. Subjects may refuse or withdraw at any

time and will not be treated unfavorably or lose any benefits that they would have received if they had

not participated in this clinical trial.

1. Information that may influence the subject's decision to continue participation in this study is promptly

communicated to the subject when it becomes available.

1. Conditions or reasons for discontinuation of participation in the trial.
2. The monitors, auditors, clinical review committee and regulatory authorities must have access to the

source documents related to the medical treatment. In such cases, the confidentiality of the subject

should be maintained. In addition, the subject's name and seal or signature on the consent form should be required to authorize access.

1. Sources of funding and conflicts of interest for conducting clinical trials
2. Description of money paid to subjects.
3. If the subject is required to bear the costs, the details.
4. Name, title and contact details of the principal investigator and sub-investigator.
5. Contact person for enquiries and emergencies.
6. Matters to be observed by test subjects.
7. Matters related to the Investigational Review Committee, including the Investigational Review

Committee that investigates and deliberates on the appropriateness of the study and other matters to be

investigated and deliberated on.

# Investigational drugs

## Name of investigational drug

Investigational drug code name: AJA030

Active drug: L-arginine (ARGI-U^®^ Combination granules)

Placebo: Preparations that do not contain L-arginine and are visually indistinguishable from the active drug.

## Composition, properties and method of preparation

### Composition

The composition of the active drug is shown in Table 5.2.1.

Table 5.2.1 Composition of the active drug

| Ingredients in 1.3 g of active drug | |
| --- | --- |
| L-arginine content | additive |
| L-arginine hydrochloride 605 mg.  L-arginine 500 mg.  (1000 mg in total as L-arginine). | calcium carmellose  hydroxypropyl cellulose  crystalline cellulose |

### Properties

White granule formulation, odourless, with a slight peculiar taste.

## Containers, packaging, labelling and storage conditions

(1) Containers and packaging

(1) The investigational drug manufactured are packaged in 2-sheet packaging, 50 sheets are placed in one small box (100 packets) and seven of these small boxes are placed in one case box (700 packets).

　(ii) For one case (3700 packets), 5 case boxes (3500 packets) + 2 small boxes (200 packets) and put them in one large box (3700 packets).

(2) Indication.

Small boxes and case boxes should be labelled to indicate that they are for investigational use, the name, title and address of the coordinating investigator, the name of the investigational drug, serial number, method of storage and expiry date.

　(i) Small box labelling (sample)

For Clinical Trials

Investigational drug name: AJA030 (100 packets)

　 Study protocol number: AJA030-002 Production number: ●●●●

　 Storage method: room temperature (1-30°C) Expiry date: ○○○○

Coordinating Investigator:

Osamu Onodera, Professor, Department of Neurology, Niigata University Medical and Dental Hospital

1-754 Asahimachidori, Chuo-ku, Niigata City, Niigata Prefecture TEL025-223-6161

　(ii) Case box labeling for allocation (sample).

For Clinical Trials　 Assignment number:

Investigational drug name: AJA030 (700 packets)

　 Study protocol number: AJA030-002 Production number: ●●●●

　 Storage method: room temperature (1-30°C) Expiry date: ○○○○

Please:

▪ This investigational medicinal product should not be used on more than one subject.

▪ Do not discard used boxes, unused investigational drugs and boxes until the end of the trial.

Coordinating Investigator:

Osamu Onodera, Professor, Department of Neurology, Niigata University Medical and Dental Hospital

1-754 Asahimachidori, Chuo-ku, Niigata City, Niigata Prefecture TEL025-223-6161

　(iii) Labeling of small boxes for allocation (sample).

For Clinical Trials　 Assignment number:

Investigational drug name: AJA030 (100 packets)

Study protocol number: AJA030-002 Production number: ●●●●

Storage method: room temperature (1-30°C) Expiry date: ○○○○

Please:

∙ This investigational drug should not be used on more than one subject.

∙ Do not discard used boxes, unused investigational drugs and boxes until the end of the trial.

Clinical trial co-ordinating physician:

Osamu Onodera, Professor, Department of Neurology, Niigata University Medical and Dental Hospital

1-754 Asahimachidori, Chuo-ku, Niigata City, Niigata Prefecture TEL025-223-6161

(iv) Large box labeling for allocation (sample)

For Clinical Trials　 Assignment number:

Investigational drug name: AJA030 (3700 packets)

　 Study protocol number: AJA030-002 Production number: ●●●●

　 Storage method: room temperature (1-30°C) Expiry date: ○○○○

Please:

∙ This investigational drug should not be used on more than one subject.

∙ Do not discard used boxes, unused investigational drugs and boxes until the end of the trial.

Coordinating Investigator:

Osamu Onodera, Professor, Department of Neurology, Niigata University Medical and Dental Hospital

1-754 Asahimachidori, Chuo-ku, Niigata City, Niigata Prefecture TEL025-223-6161

(3) Storage conditions.

Storage at room temperature (1-30°C)

## Storage of investigational medicinal products

The investigational drug manager appropriately stores and manages the investigational medicinal product in accordance with the 'Procedures for the management of investigational drugs' established by the investigational coordinator, and prepares an investigational drug management chart to ascertain the status of the use of the investigational drug and to check the receipt and delivery status.

# Methods of clinical trials

## Type of clinical trial design

The study will be conducted in a placebo-controlled, double-blind, randomised, group-controlled study. There will be two study groups: the active drug group and the placebo group, with 20 patients allocated to each group.

### Rationale for setting up the trial design.

The trial design is a double-blind, randomised, group-comparison study with placebo as the control and random allocation and blinding between the actual drugs. This design is often used in phase II trials and is considered suitable for assessing the efficacy and safety of the original real drug, eliminating the placebo effect.

## Target number of cases

Target number of patients: 40 (20 in the active drug group and 20 in the placebo group)

### Basis for setting the target number of cases

The target number of patients was set at 20 in each group, for a total of 40 patients. As the trial is positioned as a pilot study to explore and evaluate the efficacy and safety of the investigational drug administered for the first time to the patients concerned, this was set as the minimum number of patients required in consideration of feasibility.

## Procedures for conducting clinical trials

### Procedure for obtaining consent and randomising subjects.

(1) Informed consent.

Informed consent to participate in the trial in accordance with section 6.1 of this protocol.

(2) Assignment of subject identification codes.

The investigator or sub-investigator assigns a subject identification code to each subject for whom consent to participate in the trial has been obtained. Subject identification codes are given in the order described. This subject identification code will be used throughout the clinical trial.

(3) Screening

Pre-registration testing is carried out on those for whom written consent to participate has been obtained. Subjects who meet all Inclusion criteria and none of the exclusion criteria as a result of the pre-enrolment test are enrolled according to the procedure described in the next section '(4). Enrolment' in the following section.

(4) Registration.

The investigator or sub-investigator accesses the registration system and enters the necessary information for registration. After eligibility is verified in the registration system, eligible cases are assigned a registration number.

(5) Maintain random allocation and blinding of investigational drugs

(i) The assignment manager should prepare and keep an investigational drug allocation chart.

(ii) The assignment manager should check the indistinguishability of the active drug and placebo in allocating investigational drug.

(iii) The assignment manager allocates the investigational drug according to the allocation table.

(iv) The assignment manager should seal and store the allocation sheet in an envelope after the investigational drug has been allocated.

(v) The assignment manager should prepare an emergency key code and request the investigational coordinator to keep it.

(vi) If the clinical trial coordinator decides that a serious adverse event has occurred and an emergency keycode opening is necessary based on a report from the investigator, he/she opens the keycode for the relevant case and notifies the investigator.

(vii) Prior to opening the key, the investigator responsible for allocating the investigational drug should check that blindness is maintained (no key codes other than the emergency key code that was opened have been broken) and that all data are fixed.

### Investigational drug administration

The investigational drug (active drug or placebo) is packaged in polyethylene- aluminium foil laminate packets of 3 g each. The dose of the incestigational drug is 0.5 g per kg body weight as granules, administered orally in three divided doses per day for 48 consecutive weeks. The dose is determined according to body weight at the time of the pre-enrolment examination in accordance with Table 8.3.2 'Dosage Conversion Table by Body Weight', with the maximum dose at a body weight of 70 kg (11 packets/day). The investigational drug should be taken at approximately the same time of the day, morning, noon and evening. Investigational drug can be taken with anything other than water or with meals/snacks. Subjects should record their medication in a daily medication diary. The first oral dose of the investigational drug should be administered after the medical examination on Day 0, under the watchful eye of the investigator (subinvestigator) or collaborator.

If the investigational drug is vomited or spilled due to vomiting, do not take another dose as a replacement for it. If a dose cannot be taken at the scheduled dosing time due to a missed dose or a hospital visit, do not double the dose the next time, but take the scheduled single dose as soon as it becomes available, followed by an interval of at least two hours before resuming the next dose.

At the visit to collect the blood level measurement sample, the subject must be instructed to allow at least 6 hours between taking the investigational drug and collecting the sample. At the morning visit, do not take the study medication before or after breakfast. At the afternoon visit, do not take the incestigational drug before or after lunch. Any investigational medicines skipped for blood concentration measurement should be submitted to the investigator or collaborator as leftover medicines at the time of the visit. There is no need to take them together at the next oral dose.

Table 6.3.2 Internal dose conversion table by body weight （in Japanese）

### Basis for setting doses.

The active drug is covered by insurance for congenital urea cycle disorders and lysinuric protein intolerance as ARGI-U^®^ combination granules, with doses indicated up to 0.5 g/kg/day (0.384 g/kg/day as L-arginine).

On the other hand, in pharmacological studies, efficacy was observed in SCA1 knock-in mice with oral administration of 6% L-arginine hydrochloride (Fig. 8.3.3a), and in subsequent studies to determine the effective concentration range, an improvement trend was observed with administration of 4% and 6% L-arginine (Fig. 8.3.3b). Furthermore, a partial efficacy was also observed with 2% L-arginine hydrochloride (Fig. 8.3.3c).

Figure 8.3.3a. therapeutic effect of oral administration of L-arginine hydrochloride (6%) on SCA1 knock-in mice.


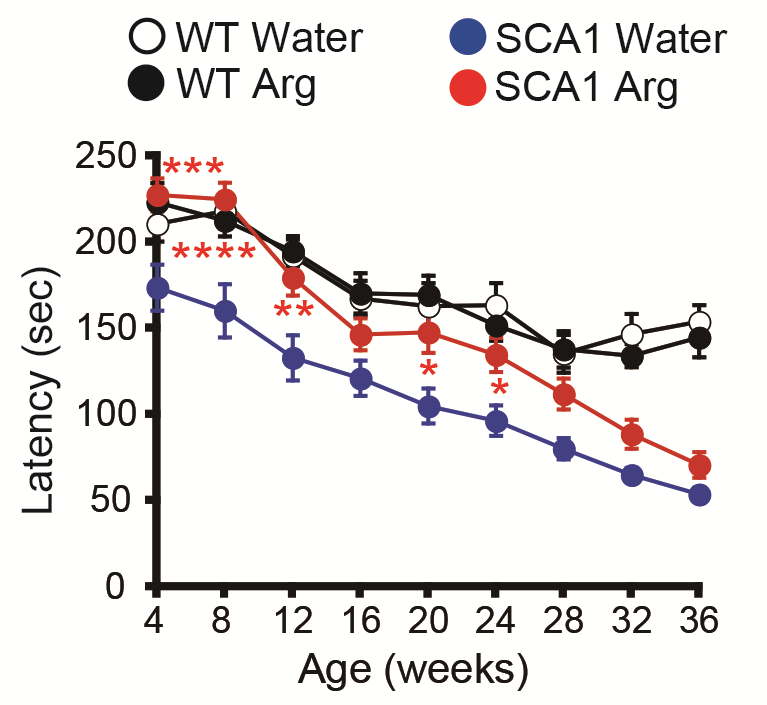


SCA1 knock-in or wild-type mice were orally administered L-arginine hydrochloride (6%) or water from 3 weeks of age and locomotor function was assessed in the rotarod test. The results showed that oral administration of L-arginine hydrochloride (6%) significantly improved locomotor disturbance in SCA1 knock-in mice.

Fig. 8.3.3b. Therapeutic effect of oral administration of L-arginine hydrochloride (6%) on SCA1 knock-in mice.


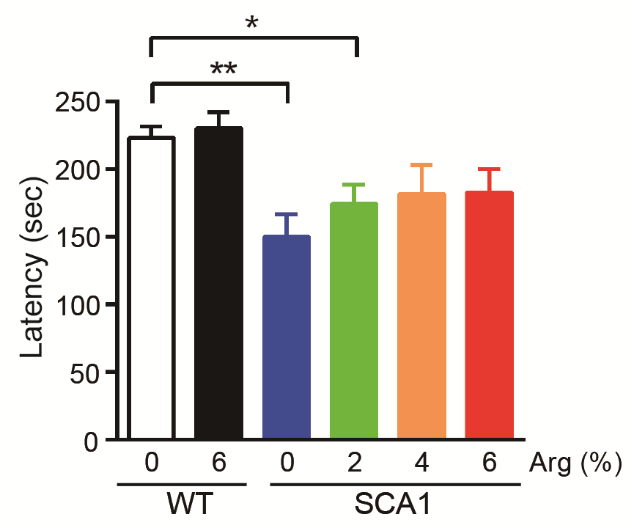


SCA1 knock-in or wild-type mice were orally administered L-arginine hydrochloride (2-6%) or water from the age of 3 weeks and their locomotor function was assessed in a rotarod test (4 weeks). The results showed that oral administration of L-arginine hydrochloride (4-6%) tended to improve locomotor deficits in SCA1 knock-in mice.

Fig. 8.3.3c. Effect of treatment with oral L-arginine hydrochloride (2%) from post-onset SCA1 knock-in mice.


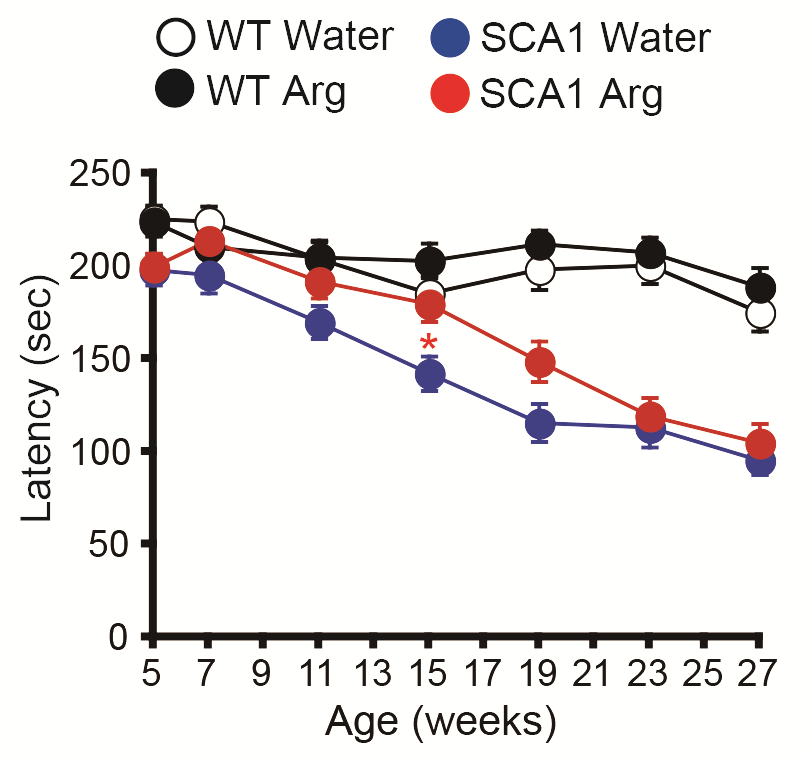


L-arginine hydrochloride (2%) or water was orally administered to SCA1 knock-in mice from 5 weeks after the onset of locomotor disturbance and locomotor function was assessed in the rotarod test. The results showed that oral administration of L-arginine hydrochloride (2%) from after the onset of SCA1 knock-in mice also resulted in significant improvement of locomotor disturbance.

Human equivalent doses based on FDA criteria for L-arginine doses to mice correspond to approximately 0.27 g/kg/day (2%), 0.54 g/kg/day (4%) and 0.80 g/kg/day (6%) (as ARGI-U^®^ combination granules, approximately 0.35, 0.7 and 1.0 g/kg/day respectively). At a dose of 0.5 g/kg/day of ARGI-U^®^ combination granules, a test subject weighing 60 kg would take 30 g/day of formulated granules (10 g at a time as three divided doses). Based on the results of animal studies, higher doses (0.7~1.0 g/kg/day) may be expected to have a higher effect.

In terms of safety, the drug is a water-soluble amino acid preparation and is considered to be virtually harmless to the human body. Furthermore, for congenital urea cycle disorders and lysinuric protein intolerance, the dose of ARGI-U ^®^ combination granules is 0.5 g/kg/day and may be increased or decreased as appropriate. However, there are no data on the safety of long-term use of L-arginine 0.8 g/kg/day (40 g at 50 kg), and when a single dose of L-arginine 0.8 g/kg/day equivalent to 50 kg was taken in volunteers in a pilot study, diarrhoea symptoms occurred at a high frequency. As this is a long-term study, it is important to ensure safety and tolerability. Therefore, a dose of 0.384 g/kg/day of L-arginine (0.5 g/kg/day as ARGI-U^®^ Combination Granules), which is assumed to be effective and has been well tolerated in other diseases, was set.

### Rationale for setting the duration of administration

Traditionally, treatment intervention trials in spinocerebellar degeneration have used the SARA (the Scale for the Assessment and Rating of Ataxia) total score as the primary endpoint, often for a treatment period of 8 to 48 weeks (12 months) (2-5,31-34) . SARA annual changes in natural history have been reported to range from 1.3 to 1.6 for SCA6 and 1.1 to 1.6 for MJD/SCA3 (35-38) . The current investigational drug suppresses polyglutamatergic pathology and does not achieve a dramatic improvement in symptoms; when SARA is the primary endpoint, a somewhat longer observation period is required to determine the effect of treatment on the suppression of progression of slight progression of symptoms as a therapeutic effect. Therefore, based on the results of clinical trials conducted to date with other drugs in spinocerebellar degeneration, the duration of treatment in the clinical trials was set at 12 months.

## Assessment of safety and efficacy

### Safety assessment

Vital signs (temperature, blood pressure, pulse), body weight, adverse events, haematology, blood biochemistry and urinalysis are performed before enrolment, on the first day of treatment, 4, 8, 16, 24, 32, 40, 48 and 52 weeks after treatment to assess safety.

### Efficacy primary endpoint

Change from baseline in SARA 'total' score at 48 weeks post-treatment.

### Basis for establishing efficacy primary endpoint.

In general, cerebellar degeneration due to SCD begins in the cerebellar vermis, which is involved in postural control, and spreads to the cerebellar hemispheres, which are involved in the control of coordinated limb movements. The SARA (the Scale for the Assessment and Rating of Ataxia) is a comprehensive measure of cerebellar ataxia in the limbs and trunk. SARA is a comprehensive measure of cerebellar ataxia in the limbs and trunk. To date, SCD trials have often used the SARA total score for the purpose of an overall assessment, which was also used for comparability with other drugs. As it is considered clinically meaningful for an investigational drug to improve the SARA total score even slightly compared to placebo, the amount of change in the SARA total score during the treatment period was used.

### Efficacy secondary endpoints

The following items are assessed as secondary items.

1. SARA 'total' scores after 4, 8, 16, 24, 32, 40 and 48 weeks
2. SARA 'walking, standing' scores after 4, 8, 16, 24, 32, 40 and 48 weeks
3. SARA scores after 4, 8, 16, 24, 32, 40 and 48 weeks for each item

(excluding walking and standing scores)

1. 48 weeks later Total trajectory length, rectangular area for 30 s in the gravity body sway test
2. Minimum time for Timed Up and Go Test (TUG) after 48 weeks
3. Beck Depression Inventory-II (BDI-II) total score at 48 weeks
4. Clinical Global Impression after 4, 8, 16, 24, 32, 40 and 48 weeks - Clinical Global Impression

Severity (CGI-S)

1. Clinical Global Impression after 4, 8, 16, 24, 32, 40 and 48 weeks - Clinical Global Impression

Improvement (CGI-I)

1. Patient Global Impression after 4, 8, 16, 24, 32, 40 and 48 weeks - Patient Global Impression

Improvement (PGI-I)

1. Short-Form 8 (SF-8) total score after 48 weeks

## Observations/test items and trial implementation schedule

### Schedule of clinical trials

　The clinical trial schedule is shown in Table 8.5.1.

Table 8.5.1 Clinical trial schedule

| **Obserrvations** | **Informed Consent** | **Screening** | **Registration/**  **Assignment** | **Duration of investigational drug administration^#6^** | | | | | | | | **Post-observation period** | **Time of discontinuance** |
| --- | --- | --- | --- | --- | --- | --- | --- | --- | --- | --- | --- | --- | --- |
|  |  |  |  | 0th day | 4 weeks | 8 weeks | 16 weeks | 24 weeks | 32 weeks | 40 weeks | 48 weeks | 52 weeks |  |
| Scheduling window | Within 4 weeks prior to registration | | 7 days prior to registration to before  the start of administration | Start Day | ±5 days | ±7 days | ±7 days | ±7 days | ±7 days | ±7 days | ±7 days | ±7 days | - |
| Obtaining consent | ○ |  |  |  |  |  |  |  |  |  |  |  |  |
| Eligibility check | ○ |  | ○ |  |  |  |  |  |  |  |  |  |  |
| Registration and allocation |  |  | ○ |  |  |  |  |  |  |  |  |  |  |
| Administration of an investigational drug |  |  |  |  |  |  |  |  |  |  |  |  | (Check status of internal medication). |
| Subject background |  | ○ |  |  |  |  |  |  |  |  |  |  |  |
| Height |  | ○ |  |  |  |  |  |  |  |  |  |  |  |
| Weight |  | ○ |  | ○^#2^ |  |  |  |  |  |  | ○ | ○ | ○ |
| Vital signs |  | ○ |  | ○^#2^ | ○ | ○ | ○ | ○ | ○ | ○ | ○ | ○ | ○ |
| Distribution of medication logbook |  |  |  | ○ | ○ | ○ | ○ | ○ | ○ | ○ |  |  |  |
| Collection and verification of medication logbook |  |  |  |  | ○ | ○ | ○ | ○ | ○ | ○ | ○ |  | ○ |
| Hematology |  | ○ |  | ○^#2^ | ○ | ○ | ○ | ○ | ○ | ○ | ○ | ○ | <○> |
| Biochemistry |  | ○ |  | ○^#2^ | ○ | ○ | ○ | ○ | ○ | ○ | ○ | ○ | <○> |
| Urinalysis |  | ○ |  | ○^#2^ | ○ | ○ | ○ | ○ | ○ | ○ | ○ | ○ | <○> |
| Pregnancy Test #1 |  | ○ |  | ○^#2^ |  |  |  |  |  |  |  |  |  |
| SARA |  | ○ |  | ○^#2^ | ○ | ○ | ○ | ○ | ○ | ○ | ○ | ○ | <○> |
| Gravity body Sway Test ^#3^ |  | ○ |  | ○^#2^ |  |  |  |  |  |  | ○ | ○ |  |
| TUG |  | ○ |  | ○^#2^ |  |  |  |  |  |  | ○ | ○ | <○> |
| BDI-II |  | ○ |  | ○^#2^ |  |  |  |  |  |  | ○ | ○ | <○> |
| CGI-S |  | ○ |  | ○^#2^ | ○ | ○ | ○ | ○ | ○ | ○ | ○ | ○ | <○> |
| CGI-I |  |  |  |  | ○ | ○ | ○ | ○ | ○ | ○ | ○ | ○ | <○> |
| PGI-I |  |  |  |  | ○ | ○ | ○ | ○ | ○ | ○ | ○ | ○ | <○> |
| SF-8 |  | ○ |  | ○^#2^ |  |  |  |  |  |  | ○ | ○ | <○> |
| MMSE |  | ○ |  | ○^#2^ |  |  |  |  |  |  | ○ |  | <○> |
| C-SSRS |  | 〇 |  |  |  |  |  |  |  |  |  | 〇 | <○> |
| AJA030 PK blood sampling ^#4^ |  |  |  | ○ | ○ |  |  | 〇 |  |  | ○ | ○ | <○> |
| Identification of adverse events^#5^ |  |  |  |  |  |  |  |  |  |  |  |  |  |
| Concomitant medications and therapies |  |  |  |  |  |  |  |  |  |  |  |  |  |

#1: Performed in female subjects of childbearing potential (if pregnancy test is performed with blood, draw approximately 2 mL more blood separately).

#2: If data from pre-registration testing is within one week of the start date of administration, the data may be adopted.

#3: Only cases from Niigata University Medical and Dental Hospital should be conducted.

#4: At the visit where the blood level measurement sample is collected, the subject must be instructed to allow at least 6 hours between taking the investigational drug and collecting the sample. At the morning visit, do not take study medication before or after breakfast. At the afternoon visit, do not take the investigational drug before or after lunch. Any investigational medicines skipped for blood concentration measurement should be submitted to the investigator or collaborator as leftover medicines at the time of the visit. There is no need to take them together at the next oral dose.

#5: Adverse event assessments at the start date of dosing are carried out after the administration of theinvestigational drug.

#6: Weeks 12, 20, 28, 36 and 44 will be telephoned by the site's clinical trial staff to check status and to confirm internal medication status. Tolerance is ±7 days.

<○>: If the investigational drug is discontinued during the investigational drug administration period, it will be carried out as far as possible.

### Observations/test items

1. **Obtaining consent**

Record the date consent was obtained.

1. **Subject background**
2. Observation period: pre-registration

2) Observations: date of birth, gender, current medical history, complications

1. **Height**

1) Observation period: pre-registration

2) Observations: height

Measure without footwear or prosthetics.

1. **Body weight**

1) Observation period: pre-enrolment, start date, 48 and 52 weeks, and at discontinuation

2) Observations: body weight

1. **Vital signs**

1) Observation period: pre-enrolment, at start of treatment, 4, 8, 16, 24, 32, 40, 48 and 52 weeks, and at discontinuation

2) Observations: body temperature, blood pressure (systolic and diastolic), pulse

Resting blood pressure and pulse rate are measured in a sitting position. Measurements should be taken using the same arm (usually the arm opposite the dominant arm) and an appropriately sized cuff.

1. **Medication log**

1) Observation period: starting date, 4, 8, 16, 24, 32, 40 and 48 weeks, and at discontinuation

2) Observations: medication records

Distribution, collection and verification of medication diaries.

1. **Haematological examination**

1) Observation periods: pre-enrolment, start date, 4, 8, 16, 24, 32, 40, 48 and 52 weeks, (at *discontinuation)

2) Observations: red blood cell count, white blood cell count, white blood cell fractions (neutrophils, lymphocytes, monocytes, eosinophils, basophils) platelet count, haemoglobin, haematocrit values

1. **Blood biochemical tests**

1) Observation periods: pre-enrolment, start date, 4, 8, 16, 24, 32, 40, 48 and 52 weeks, (at *discontinuation)

2) Observations: AST, ALT, ALP, γGTP, LDH, CPK, serum creatinine, Na, K, Ca, Cl, P, BUN, triglycerides, total cholesterol, blood sugar at any time, CRP

1. **Urinalysis**

1) Observation periods: pre-enrolment, start date, 4, 8, 16, 24, 32, 40, 48 and 52 weeks, (at *discontinuation)

2) Observations: occult blood, protein, sugar, urobilinogen

1. **Pregnancy tests (performed only on female subjects of childbearing potential).**

1) Time of observation: pre-enrolment, date of administration

2) Observations: pregnancy test (urine hCG or serum hCG)

1. **Evaluation of SARA scores.**

1) Observation periods: pre-enrolment, start date, 4, 8, 16, 24, 32, 40, 48 and 52 weeks, (at *discontinuation)

2) Observations: SARA score (gait, standing, sitting, speech impairment, finger tracking test, nose-to-finger test, hand gyration/rotation movements, heel-shin test) Assessment.

　　　　　　　　　The SARA score assessment is performed by another investigator who is not the subject's investigator. The assessor is also prohibited from conversing with the subject about the medications.

1. **Gravity body sway test**

1) Observation periods: pre-enrolment, start date, 48 and 52 weeks

2) Observations: Gravity body sway test

Only at Niigata University Medical and Dental Hospital.

1. **Timed Up and Go Test (TUG)**

1) Observation periods: pre-enrolment, start date, 48 and 52 weeks, (*discontinuation)

2) Observations: Timed Up and Go Test

Perform one at normal walking speed and one at maximum walking speed, with the value of the maximum walking speed being the measured value.

1. **BDI-II Assessment.**

1) Observation periods: pre-enrolment, start date, 48 and 52 weeks, (*discontinuation)

2) Observations: BDI-II assessment

1. **CGI Rating**

1) Observation periods: pre-enrolment, start date, 4, 8, 16, 24, 32, 40, 48 and 52 weeks, (at *discontinuation)

2) Observations: CGI-S (severity) and CGI-I (improvement) assessments

Before enrolment and on the day of dosing, only CGI-S assessments are carried out.

1. **PGI-I assessment**

1) Observation period: 4, 8, 16, 24, 32, 40, 48 and 52 weeks, (*on discontinuation)

2) Observations: PGI-I assessment

1. **SF-8 evaluation**

1) Observation period: pre-enrolment, start date, 48 and 52 weeks, (*discontinuation)

2) Observations: SF-8 assessment

1. **MMSE evaluation**

　 1) Observation period: pre-enrolment, start date, 48 weeks, (*discontinuation)

2) Observations: MMSE assessment

1. **C-SSRS Assessment**

1) Observation period: pre-enrolment, 52 weeks, (*on discontinuation)

2) Observations: C-SSRS assessment

1. **Measurement of plasma arginine concentration (plasma amino acid analysis)**

1) Observation period: start date, 4, 24, 48 and 52 weeks, (at *discontinuation)

2) Observations: blood samples are taken for measurement of plasma arginine concentration (SRL amino acid analysis [9 species]).

At the visit to collect the blood sample, the subject should be instructed to allow at least 6 hours between taking the investigational drug and collecting the sample. The site staff should confirm with the subject the date and time of the most recent dose of investigational drug prior to collection of the blood sample. Record the date and time of collection of the blood concentration specimen. The date on the case report form should be clearly recorded (e.g. DD MM YYYYY) and the time of collection should be recorded to the minute (HH:MM, 24-hour format).

1. **Identification of adverse events**

1) Observation period: starting date, 4, 8, 16, 24, 32, 40, 48 and 52 weeks, and at discontinuation

2) Observations: name of adverse event, date of onset, severity, severity, causal relationship to investigational, treatment, outcome and date of outcome

1. **Check concomitant medications and concomitant therapies**

1) Observation period: pre-enrolment, at start of treatment, 4, 8, 16, 24, 32, 40, 48 and 52 weeks, and at discontinuation

2) Observations: concomitant medications

Record the drug name (generic name), route of administration, dose, duration of administration and reason for concomitant use.
 Concomitant therapy

Record the name of the treatment, date and reason for implementation.

　(* when cancelled) should be carried out as far as possible, and the absence does not constitute a deviation from the plan.

If the data from the pre-registration test is within one week of the start date of administration, the data from the pre-registration test may be adopted as the data for the start date of the investigational drug administration.

# Management of test subjects.

## Health care

If a subject receives treatment from a physician other than the investigators and subinvestigators of the trial, notify the physician concerned of the subject's participation in the trial by having the subject present a clinical trial participation card, and instruct the subject to contact or enquire with the investigators as necessary.

## Prohibited drugs and drug-free treatment

All concomitant medications (including Seresist^🄬^ (tartirelin hydrate) and Hyltonin Injection^🄬^ (protirelin tartrate hydrate)) are permitted during the study period. However, the dose should be kept as constant as possible for 4 weeks prior to the start of investigational drug administration. For Seresist^🄬^ (tartirelin hydrate), Hyltonin Injection^🄬^ (protilerin tartrate hydrate) and phosphodiesterase 5 inhibitors, initiation or discontinuation during a clinical trial is not permitted and any initiation or discontinuation is treated as a drop-out or discontinuation case. The investigator (sub-investigator) or collaborator should be instructed to promptly inform the investigator (sub-investigator) or collaborator of any changes in concomitant medication.

Rehabilitation started at the time of enrolment in the clinical trial will continue without any change in content. No new rehabilitation will be initiated throughout the duration of the clinical trial.

## Follow-up after completion or interruption of the clinical trial (when necessary)

Undertake follow-up as necessary on adverse events observed during the trial period, in accordance with the instructions of the investigator or sub-investigator.

# Ensuring the safety of test subjects

## Ensuring the safety of subjects

The investigators and sub-investigators should comply with the following basic requirements to ensure the safety of the subjects

(i) The investigators and sub-investigators should comply with this protocol.

(ii) The investigators and sub-investigators should endeavour to detect adverse events at an early stage, and if they consider that an adverse event has occurred and treatment is necessary for the subject, they should inform the subject and provide appropriate medical treatment. (iii) The investigators and sub-investigators should discontinue the clinical trial for the subject concerned depending on the severity of the adverse event in accordance with section 10.2 of this protocol.

(iii) If the investigators and subinvestigators receive new safety or adverse effect information related to the investigational drug that may affect subject participation, they will promptly communicate this information to the subject in accordance with section 11.6 of this protocol.

## Criteria for discontinuation of a clinical trial per subject.

The investigator or sub-investigator sh discontinue the subject's clinical trial in the following cases

1. If the subject withdraws consent to participate in the clinical trial

If consent is withdrawn for the use of all data, a consent withdrawal form should in principle be used, except in cases where it is difficult to come to the hospital. If only some of the survey items are withdrawn, a consent withdrawal form is not required and this should be recorded in the source documents.

1. When it is deemed difficult to continue the administration of the investigational drug to the subject concerned due to adverse events, etc.
2. Treatment that is deemed to have a significant impact on the outcome of the trial, such as the administration of surgical treatment during the trial period.
3. If a female subject becomes pregnant during the trial (if the male subject's partner becomes pregnant, the trial can continue)
4. If it is found after registration that the inclusion/exclusion criteria are violated.
5. If the subject is unable to continue the clinical trial for non-medical reasons (e.g. hospital transfer, relocation, etc.)
6. Other cases where the investigator or sub-investigator decides that it is difficult to continue the trial.

## Procedure for discontinuation of a clinical trial per subject.

The investigator decides whether the subject can continue the trial and, if not possible, follows the procedure for discontinuation.

- - - 1. The investigator or subinvestigator should promptly explain the discontinuation of the clinical trial to

the subject concerned and the surrogate if any, and take necessary measures such as provision of

appropriate medical care and guidance on health management.

2) Information on subjects collected in accordance with the study protocol before discontinuation of the study will be analyzed in accordance with section 13 of the study protocol.

3)　For subjects who have received an investigational drug, check safety after discontinuation of the investigational drug.

# Adverse event

## Definition of adverse events.

An 'adverse event' is any unwanted or unintended sign (including abnormal laboratory values), symptom or disease that occurs in a subject to whom an investigational drug has been administered, regardless of whether or not it is causally related to the investigational drug. Signs, symptoms or diseases that were present before the investigational drug was administered and that worsen afterwards are also considered adverse events, but signs, symptoms or diseases that were present before consent to participate in the trial and that do not worsen significantly, or physiological changes that are not considered clinically significant in terms of frequency or severity of occurrence, are not considered adverse events.

Among adverse events, those for which a causal relationship with the investigational drug cannot be denied, i.e. those events in "Events with a causal relationship in Table 11.2.3", are referred to as "adverse reactions". An adverse event is said to be an 'unanticipated adverse event' if the adverse event is not described in the investigational new drug summary, or if it is described, but the number, frequency, conditions of occurrence, severity and other trends of occurrence do not match those described. Adverse events that meet the definition in '9.2.5 Severity of adverse events' are also referred to as 'serious adverse events'.

## Assessment of adverse events

The investigator or sub-investigator should enter the following information in the case report form for all adverse events observed during the trial. If more than one adverse event is observed, enter each event separately.

1. Name of adverse event
2. date of appearance
3. Severity (see 9.2.2)
4. Severity (non-serious and serious)
5. Causal relationship with investigational drug
6. treatment
7. Outcome (recovered, mildly recovered, not recovered, recovered but with sequelae, dead, unknown) and date of outcome

### Determination of adverse events

　Abnormal laboratory values or vital signs that are accompanied by clinical symptoms, require interruption or discontinuation of the investigational drug, require medical intervention such as changing concomitant medications, or are judged to be clinically significant findings by the investigator or sub-investigator should be reported as adverse events.

### Severity of adverse events.

The investigator or sub-investigator assesses the severity of the adverse event according to the following criteria.

Mild: usually transient, does not impair the subject's daily life and does not require treatment (normal activities are possible)

Moderate: to the extent that it interferes somewhat with the subject's daily life, causes sufficient discomfort and requires treatment (with discomfort in activities).

Severe: to the extent that the subject's performance of daily activities is significantly impaired and requires treatment (difficulty in performing normal activities).

### Causal relationship between investigational drug and adverse events.

The assessment of a causal relationship between the investigational drug and the adverse event is performed by the investigator or sub-investigator according to Table 11.2.3. If a causal relationship between the investigational drug and the adverse event cannot be ruled out, the assessment is 'causal'.

Table 9.2.3 Assessment of the causal relationship between the investigational drug and the adverse event

| Assessment of causality | causally related  (where a causal link cannot be ruled out). |
| --- | --- |
|  | No causal relationship |

### Predictability of adverse events.

Adverse events that cannot be predicted from the investigational drug summary of the investigational drug in question (including adverse effects that have already been reported to the Minister of Health, Labour and Welfare by the investigator), such as the occurrence of such events, etc. or the trend of occurrence, including the number of occurrences, frequency of occurrences and conditions of occurrence, should be classified as "unpredictable" or "unknown" and predictable as "predictable" or "known". The following should be used.

### Severity of adverse events

Serious adverse events are defined as any of the following, regardless of the severity of the adverse event.

- - 1. death
    2. Threatening to cause death.
    3. Admission to a hospital or clinic for treatment^*^ or extended hospital stay required
    4. disability
    5. Items that may lead to disability.
    6. (i) Those that are serious according to (i) to (v).
    7. Congenital diseases or anomalies in later generations

*In this clinical trial, 'hospitalisation' means staying at a medical institution for one or more nights to receive treatment, and does not include prolonged outpatient treatment such as intravenous infusion or hospitalisation for the purpose of tests. In the case of prolonged treatment in the emergency department, the investigator or sub-investigator will consider the situation and decide whether it should be regarded as a hospitalisation.

## Main adverse events and reactions expected in this trial

According to clinical trials and post-marketing use-results surveys of ARGI-U ^®^ combination granules, nausea, nausea, vomiting, diarrhoea, scratchiness and abnormal liver function were reported as the main adverse reactions.

Care should be taken throughout this clinical trial.

### Major adverse events foreseen in this trial

1. Nausea, nausea and vomiting
2. Diarrhoea
3. Feeling that one's skin is itchy
4. Abnormal liver function (increased AST, ALT, ALP)

The frequency of occurrence, calculated from the combined results of clinical trials and post-marketing surveillance up to the time of approval, ranged from 0.1% to less than 5% in all cases.

## Response to adverse events.

### Dealing with anticipated adverse events.

In the event of an adverse event, the investigators and sub-investigators should provide appropriate first aid, pay attention to ensuring the safety of the subject, and endeavour to resolve and investigate the cause by having the subject undergo diagnosis and treatment by a specialist physician when necessary.

For each anticipated adverse event, take the following actions

1. For nausea, nausea and vomiting, careful follow-up or antiemetics should be administered according to symptoms.
2. For diarrhoea, careful follow-up or intestinal regulators should be administered according to symptoms.
3. Careful follow-up or anti-allergic agents should be administered for itching, depending on the symptoms.
4. If liver function abnormalities (increased AST, ALT, ALP) exceed three times the upper limit of the institutional reference value, the investigator (subcontractor) will inform the study coordinating investigator of the results of a thorough examination of the causal relationship with the investigational drug and discuss the need to discontinue the investigational drug.

### Follow-up of adverse events that cannot be ruled out as causally related to the investigational drug

Adverse events occurring during the trial period should be followed up as far as possible until recovery occurs or the event is deemed no longer clinically necessary. The method of inpatient or outpatient visits, or in the case of outpatients, the frequency of visits and test items, etc., should be determined by the investigator or sub-investigator, depending on the type and severity of the relevant adverse event. However, if there is a reasonable reason to terminate the investigation in the middle of the study, the investigator or sub-investigator may terminate the follow-up investigation by stating the reason in the original documents such as the medical record.

### Records of adverse events when they occur.

The investigator or sub-investigator should describe in the case report the name of the adverse event, date of onset, severity, severity (non-serious and serious), causal relationship to the investigational drug, treatment, outcome and date of outcome.

## Handling of serious adverse events.

If an adverse event occurs in a subject receiving an investigational drug and the investigator or others consider the event to be serious as per section 11.2.5, the adverse event information should be handled according to the following procedure.

Details on how to respond to the occurrence of a serious adverse event are in accordance with the Procedures for Handling Safety Information.

1) Reporting from the investigator to the head of the site, the clinical trial coordinator and the investigational drug provider

The investigator should report such adverse event information as soon as possible to the head of the investigational medical institution, regardless of causal relationship, as well as to the trial co-ordinator and the investigational drug provider.

2) Notification by the clinical trial coordinator to the investigators at each site

The clinical trial co-ordinator reviews the content of the adverse event report obtained from the investigator and notifies the relevant adverse event information to the investigators at each of the other sites.

3) Discussions between the investigator and the coordinating investigator

The investigator at each site checks the report obtained from the clinical trial co-ordinator, discusses it with the clinical trial co-ordinator as necessary and reports his/her opinion as the clinical trial co-ordinator (including the need for a report to the Minister of Health, Labour and Welfare) to the clinical trial co-ordinator.

4) Report to the Minister of Health, Labour and Welfare

If a report to the Minister of Health, Labour and Welfare is required, the Clinical Trial Co-ordinator should prepare Forms 7 and 8 and an organising sheet and report to the National Institute for Pharmaceuticals and Medical Devices (hereinafter referred to as 'NHI').

5) Reporting to the head of the implementing medical institution

When a report is made to the Minister of Health, Labour and Welfare on a serious adverse event that occurred at another medical institution, the investigator should report the contents of Forms 7 and 8, obtained from the clinical trial coordinator, to the head of the implementing medical institution as soon as possible.

6) Action to be taken when additional information is available.

The investigator at the site where the adverse event occurred should make an additional report to the head of the site as soon as possible when additional information on the event is obtained, as well as to the trial co-ordinator and the trial drug provider. The handling of such additional information should be in accordance with the procedures described in (1) to (5) of this section, and reports should be made to the Agency as necessary.

## Handling of safety information obtained during clinical trials.

The clinical trial coordinator will endeavour to collect information and other information (foreign case information, action reports and research reports) concerning the study's investigational drug. Details on how to respond when safety information is obtained will be in accordance with the 'Procedures for handling safety information'.

If the investigator obtains information on the quality and safety of the active drug or other information important for the proper conduct of the clinical trial, he/she prepares a 'Report on Safety Information' and reports it to the head of the implementing medical institution.

The investigator or sub-investigator should immediately explain orally to the currently participating subject any new findings that affect the subject's intention. After submitting the revised consent document to the Clinical Trial Review Committee and obtaining its approval, a new explanation document should be used to explain the new findings to the currently participating subjects and consent for continued participation should be obtained in writing again, except for subjects for whom the observation period has already ended. New findings that affect the subject's intention refer, for example, to information on new adverse events related to the treatment or information on the development of new treatments for the disease in question.

## Pregnancy and maternity and when obtaining information on childbirth

If a patient is found to be pregnant during the course of the study, the administration of the investigational drug should be discontinued immediately and recorded in the adverse event section of the source documents. The investigator or sub-investigator should follow up to the birth and report the health status of the mother and child to the head of the implementing medical institution. The reporting procedure should be in accordance with the 'Procedures for handling safety information'.

If a patient's partner is found to be pregnant, the trial will not be stopped. Pregnancy of the patient's partner is not considered an adverse event. However, if possible, the investigator or sub-investigator should follow up and record the birth.

# Effectiveness and Safety Assessment Committee

The committee consists of members with the expertise required for the evaluation of the trial and provides appropriate advice and recommendations to the trial coordinator from a neutral standpoint to ensure patient safety and the ethical and scientific validity of the conduct of the trial. The procedures of the Effectiveness and Safety Assessment Committee should follow the 'Procedures for the Effectiveness and Safety Assessment Committee' to be prepared separately.

# Ethical and scientific conduct of clinical trials

## Compliance with laws and regulations

This clinical trial should be conducted in accordance with the ethical principles of the Declaration of Helsinki, and in compliance with the Act on Quality, Efficacy and Safety Assurance of Pharmaceuticals and Medical Devices, its Enforcement Order, its Enforcement Regulations, the GCP Ministerial Ordinance, notices related to the GCP Ministerial Ordinance (hereinafter collectively referred to as 'GCP Ministerial Ordinance etc.') and this Clinical Trial Implementation Plan. The following conditions should be complied with.

## Clinical Trial Review Committee

Prior to the conduct of the trial, the Clinical Trial Review Committee deliberates on the conduct of the trial from the perspective of ethical, scientific and medical relevance. In addition, the Clinical Trial Review Committee will review any changes to the trial. If the study lasts longer than one year, the Trial Review Committee will continuously review whether the study is being conducted properly at least once a year.

## Preservation of confidentiality of test subjects

The investigators and sub-investigators should take the human rights of the subjects into consideration and endeavour to protect their personal information. Subjects should be identified by subject identification codes in the subject enrolment and case report forms, and those involved in this clinical trial should maintain the confidentiality of subjects in the direct inspection of original documents, publication in medical journals and submission of documents to regulatory authorities, etc. related to the conduct of the clinical trial.

# Statistical matters

## Definition of the analysis population

### Safety analysis population

The population of enrolled cases, excluding the following subjects, is the population for the safety analysis.

Cases not treated with investigational drug

Subjects with no safety observations at all after administration of the investigational drug

### Efficacy analysis population

The population of enrolled cases excluding the following subjects is the population subject to efficacy analysis (FAS: Full analysis set).

Cases not treated with investigational drug

Subjects with no measurements at all on the assessment of efficacy

For the primary endpoint, an analysis using a subject population (PPS: Per Protocol Set) compliant with the study protocol will also be conducted to assess the stability of the FAS analysis results.

## Criteria for handling cases

Cases should be handled as follows.

1) Ineligible cases.

Cases in breach of the 'inclusion criteria', cases in breach of the 'exclusion criteria' and cases of GCP non-compliance are ineligible cases.

2) Cases of discontinuation or drop-out prior to administration of the investigational drug

Cases of discontinuation or drop-out prior to investigational drug administration (see 3) below) are not included in the analysis population.

3) Cases of discontinuation or drop-out after administration of an investigational drug

Cases that withdrew consent, did not fulfil the 'inclusion criteria', were in conflict with the 'exclusion criteria', or were discontinued according to the criteria for discontinuation of the trial for each subject as indicated in 'Criteria for discontinuation of the trial for each subject' should be considered as discontinued cases. In addition, cases that are untraceable due to reasons not directly related to the trial should be considered as drop-out cases. These cases will be included in the analysis with the amount of change in the score at the last visit from before medication.

## Analysis items/methods

The main tabulation and analysis methods are described below. For the analysis of efficacy endpoints, analyses using various statistical methods will be performed on the data obtained, if necessary. The details of the analysis are described in a separate Statistical Analysis Plan.

### Subject background

Describe demographic variables and patient characteristics for the safety analysis population using appropriate summary statistics.

### Safety analysis

1) Analysis of adverse events and adverse events for which a causal relationship cannot be ruled out

　　For each investigational drug group (active drug group and placebo group), the number of adverse events by event (SOC and PT in the latest version of MedDRA) and by severity of the event, the number of occurrences and the percentage of occurrences (number of occurrences/number of evaluable cases) are tabulated. The same tabulation is also carried out for adverse events for which a causal relationship with the investigational drug cannot be ruled out.

2) Analysis of laboratory values

　A time trend diagram should be prepared for each subject over the entire observation period for haematological tests, blood biochemical tests and urinalysis. The number of cases and percentage of occurrence of laboratory abnormalities will be calculated.

3) Serious adverse events.

　　Narrative for each individual event.

### Analysis of efficacy endpoints

1) Primary endpoint (SARA total score after 48 weeks of treatment)

The change in SARA total score from baseline to 48 weeks post-dose is compared for the test vs control group by analysis of covariance, with baseline as the covariate and the investigational drug group (active drug group and placebo group) as the fixed effect.

2) Secondary endpoints.

SARA total score change through 48 weeks.

SARA total scores are summed for each assessment period, and the total is then subjected to a general linear mixed-effects model with the change in time (4, 8, 16, 24, 32, 36, 40 and 48 weeks) as the response variable, the investigational drug group (active drug group vs placebo group), time and baseline as fixed effects and subjects as variable effects Examine the interaction between time and investigational drug group. Multiplicity is not considered.

SARA 'walking, standing' score.

As per the method of analysis of the SARA 'total' score through 48 weeks.

SARA Scores for each item

Same analysis method for SARA 'total' scores through 48 weeks, except for the combined 'walking' and 'standing' scores for each SARA item.

Gravity body sway test

A summary of the change in total trajectory length and rectangular area (‘values after 48 weeks’ treatment’ – ‘baseline values’) obtained during the 30-second gravity body sway test is shown for each group at the time of the final assessment. Cases with a negative change are defined as improved cases, and the distribution of improved cases at the time of the final assessment is shown for each group.

TUGT.

A summary at the time of the final assessment is given for each group for the shortest time change between the two tests ('value after 48 weeks' treatment' - 'baseline value'). Cases with a negative change are defined as improved cases, and the distribution of improved cases at the time of the final assessment is shown for each group.

BDI-II.

A summary of the change in the two tests ('value after 48 weeks' treatment' - 'baseline value') at the time of the final assessment is presented for each group. Cases with a negative change are defined as improved cases, and the distribution of improved cases at the time of the final assessment is shown for each group.

CGI-S.

A summary of the amount of change in tests at each time point ('value at each time point' - 'baseline value') is presented for each group at each assessment time point. Cases with a negative amount of change are defined as improved cases, and the distribution of improved cases at each assessment time point for each group is shown.

CGI, PGI-I.

A summary at each assessment time point is given for each group.

SF-8.

A summary of the change in the two tests ('value after 48 weeks' treatment' - 'baseline value') at the time of the final assessment is presented for each group. Cases with a negative change are defined as improved cases, and the distribution of improved cases at the time of the final assessment is shown for each group.

3) Descriptive statistics, diagrams and tables

For each endpoint, calculate frequencies, proportions and 95% confidence intervals for the counts, and descriptive statistics (number of cases evaluated, mean, SD, minimum, median, maximum and 95% confidence interval) for the quantitative values, by study group and time of evaluation, as appropriate. Where necessary, drawings and tables are made.

4) Significance level

Two-sided α = 0.15 for subject background bias, one-sided α = 0.025 for the others.

### Analysis of plasma drug concentrations.

If necessary, plasma drug concentrations are analysed for data where plasma arginine concentrations are detected.

## Interim analysis

No interim analysis will be conducted in this trial.

## Procedures for preparing and modifying statistical analysis plans.

Details of the analysis items and analysis methods will be described in a separate statistical analysis plan. The statistical analysis plan should be finalised before data fixation.

If changes or additions to the analysis plan occur after the start of the trial, the appropriateness of the changes or additions and their impact on the evaluation of the trial should be considered, and the circumstances leading to the changes or additions to the analysis plan should be described in the summary report.

# Deviations from the study protocol, amendments to the study protocol, suspension or discontinuation of the entire study

## Compliance with the clinical trial protocol

The trial will be conducted in compliance with this protocol, except in medically unavoidable cases, such as when the trial is intended to avoid immediate danger to the subject.

## Deviations from the clinical trial protocol

The investigators and sub-investigators should record all deviations from the study protocol, irrespective of the reason. The investigators and subinvestigators may deviate from the protocol if there are unavoidable medical reasons, such as to avoid immediate danger to the subject. In such cases, the investigator shall report the details and reasons as soon as possible to the head of the site.

## Revision of the clinical trial protocol.

When the investigator revises the clinical trial protocol, he/she must obtain approval from the head of the implementing medical institution. If a revision of the protocol is necessary, the investigator submits it to the head of the medical institution for approval. The clinical trial review committee, which receives a request for deliberation from the head of the implementing medical institution, should thoroughly consider the ethical, scientific and medical appropriateness of the revision when making the revision.

## Suspension or discontinuation of the entire clinical trial

### Criteria for suspending or discontinuing the entire clinical trial

The investigator should discontinue or suspend the entire clinical trial in the following cases

1. When the head of the investigational institution decides that the clinical trial should not be continued

based on the report of the investigational review committee and notifies the investigator.　Serious adverse events or other serious events causally related to the investigational drug.

1. A serious event such as a serious adverse event causally related to the investigational drug occurs.
2. When new serious information is obtained that may adversely affect the safety of subjects or the

conduct of this clinical trial.

1. When the investigator finds that the site has violated the GCP ordinance or the study protocol, thereby

interfering with the proper conduct of the study.

1. If the investigator determines that the study should be terminated or cannot be continued for any other

reason.

### Procedures for suspending or discontinuing the entire clinical trial.

In case of discontinuation according to 1) of section 14.4.1) of this protocol, the investigator should stop the entry of new subjects and discontinue the ongoing subject study as soon as possible.

In case of interruption due to 2) and 3) of the same paragraph, the investigator should promptly report the interruption to the head of the institution, and the investigator should interrupt the ongoing clinical trial for the subject at the earliest possible moment, and take appropriate measures and investigate the cause as necessary.

For subjects who have received an investigational drug, check safety after discontinuation of the investigational drug.

In the event of discontinuation of a clinical trial, the investigator should promptly report the discontinuation to the head of the implementing medical institution.

# Case report

The investigators and sub-investigators should prepare case reports on all subjects enrolled in the study with regard to the pre-enrolment examinations to be performed, the administration of the investigational drug and subsequent follow-up observations.

The EDC system will be used to generate the case report forms for this trial; the EDC system will electronically record all data history as an audit trail, including who entered the form, when it was entered, what was amended and when it was amended.

# Preservation of documents or records

## Documents or records to be kept and where they are kept.

Documents or records related to the clinical trial should be stored in accordance with the administrative communication "Administrative Communication of the Drug Evaluation and Management Division, Pharmaceuticals and Consumer Health Bureau, Ministry of Health, Labour and Welfare; Regarding documents or records related to the clinical trial" (5 July 2048) notified in accordance with the GCP Ministerial Ordinance.

## Duration of strage

1) The principal investigator and the commissioned coordinator of the clinical trial.

The self-initiator and the commissioned clinical trial coordinator should retain the documents or records pertaining to the clinical trial that should be retained by the self-initiator until the later of the following (i) or (ii).

The actions to be taken after the end of the retention period should be discussed with the investigational drug provider.

(i) The date of marketing approval (or in the case of an additional indication, the date of partial marketing approval) for the investigational drug.

(or, in the case of discontinuation or notification that the trial results will not be included in the application for approval, the date on which the decision to discontinue development was made or three years have elapsed since the date on which the notification that the results will not be included in the application was received).

(ii) The date on which three years have elapsed since the discontinuation or termination of the clinical trial.

2) Medical institutions performing

(2) The person in charge of archiving materials designated by the head of the implementing medical institution should preserve documents or records pertaining to the clinical trial that should be preserved at the implementing medical institution until the later date of the following (i) or (ii). However, in cases where the person conducting the clinical trial himself/herself requires a longer retention period than this, the implementing medical institution should consult with the person conducting the clinical trial himself/herself regarding the retention period and method of retention.

(i) The date of marketing approval (or in the case of an additional indication, the date of partial marketing approval) for the investigational drug in question (the date of development).

(or, in the case of discontinuation or notification that the trial results will not be included in the application for approval, the date on which the decision to discontinue development was made or three years have elapsed since the date on which the notification that the results will not be included in the application was received).

(ii) The date on which three years have elapsed since the discontinuation or termination of the clinical

trial.

3) Investigative Review Board (IRB)

The IRB establisher should retain records such as the SOP, list of committee members (including qualifications of each member), list of occupations and affiliations of committee members, documents submitted, minutes of meetings and letters until the later date of either (i) or (ii) below. However, if the person conducting the clinical trial himself/herself requires a longer retention period than this, the retention period and method of retention should be discussed with the person conducting the clinical trial himself/herself.

(i) Date of marketing authorisation (or date of partial marketing authorisation in the case of additional indications) for the investigational drug concerned (under development).

(or the date on which the decision to cease development was made, if the applicant was notified that the results of the trial would not be included in the application for approval, or the date on which three years have elapsed since the applicant was notified that the results of the trial would not be included in the application).

(ii) The date on which three years have elapsed since the discontinuation or termination of the clinical trial.

# Quality control and quality assurance of clinical trials

## Quality control

The investigators, subinvestigators and collaborators should conduct the clinical trial in compliance with the protocol. In addition, for the purpose of standardising and controlling the quality, the investigator should prepare standard operating procedures for the conduct of the clinical trial, and the principal investigator, subinvestigators and collaborators should conduct the clinical trial in accordance with the respective procedures for the conduct of the clinical trial.

### Monitoring

Monitors confirm that the human rights, safety and welfare of the subjects are protected and that this clinical trial is conducted in compliance with the GCP ordinance, the protocol and the standard operating procedures. Monitoring is also carried out to confirm that the data and other information reported by the investigator or sub-investigator are accurate and complete by directly inspecting source documents and other clinical trial-related records. Monitors must not divulge to third parties any information on subjects obtained during monitoring.

## Quality assurance

### Data management

Management of EDC input data follows the Procedures for Data Management. Adverse events and complications should be read using the latest version of MedDRA. Drugs are read in using the ethical drug name data file. Subjects' personal information stored in the database is handled in compliance with relevant laws and regulations.

### Audit

For the purpose of quality assurance of the clinical trial, the trial co-ordinator has an auditor conduct the audit to check whether the trial is being conducted in compliance with the GCP ordinance, protocol and standard operating procedures, independently and separately from the normal monitoring and quality control operations of the trial.

# Direct access to original documents and other materials

The investigator and the site will comply with the need to make all trial-related records, including original documents, available for direct inspection during monitoring, audits, investigations by clinical review committees, regulatory authorities, etc., if this becomes necessary.

# Clinical trial costs and compensation for damage to health

## Sources of funding and conflicts of interest in this clinical trial

The costs of this clinical trial will be covered by a public research contract funded by the Japan Agency for Medical Research and Development Research Fund.

Conflicts of interest are properly managed so that no problems arise due to conflicts of interest in accordance with the separate regulations of the respective medical practices.

## Subjects' cost-sharing in relation to clinical trials

Medical costs for normal treatment during the period of participation in the study, including observation and testing, are within the scope of normal medical care and will be covered by the subject's health insurance scheme. However, the costs of investigational drugs (active drug and placebo) will be borne by the research fund of the study.

For the patient contribution reduction fee in this trial, a payment of JPY 7,000 per visit as stipulated should be made.

## Compensation for health damage

The investigator and the investigative medical institution should provide appropriate compensation if the subject suffers any health damage as a result of this clinical trial. The compensation should include the payment of medical expenses and compensation for any damage to health that occurs. The investigator should, in advance, take measures for insurance coverage, provide a medical system for treatment of side effects, etc., and take other necessary measures to compensate for any damage to the subject's health caused in relation to this clinical trial.

1) Payment of medical expenses

The investigator should provide the best possible treatment for the health hazard concerned.

Only when health problems arising from this clinical trial are treated at the hospital, compensation will be provided for the relevant health problems out of the medical costs borne by the subject.

2) Payment of compensation

Compensation is paid in the event of permanent injury or death of the subject in accordance with the indemnity liability insurance of the non-life insurance company taken out by the investigator.

# Arrangements for attribution of results and publication of trial results

Intellectual property rights arising from this clinical trial should belong to the investigator. The results of this clinical trial should be summarised in a summary report. The results of this clinical trial should also be published as a paper or conference presentation, as appropriate, after the completion of the clinical trial.

Careful consideration is given to the protection of the subject's personal data, for example, the subject's name will not be directly published when the information is made public.

# Duration of the clinical trial

Clinical trial duration: 09/09/2020 - 31/03/2023

Case registration period: 09.09.2020 - 30.09.2021

# Clinical trial implementation system

The system for conducting the clinical trial is described in Annex 1.

# References.

1. Tsuji S, Onodera O, Goto J, Nishizawa M, Study Group on Ataxic D. Sporadic ataxias in Japan--a population-based epidemiological study. Cerebellum 2008; 7:189-197

2. Di Prospero NA, Baker A, Jeffries N, Fischbeck KH. neurological effects of high-dose idebenone in patients with Friedreich's ataxia: a randomised, Lancet Neurol 2007; 6:878-886

3. Ilg W, Synofzik M, Brotz D, Burkard S, Giese MA, Schols L. Intensive coordinative training improves motor performance in degenerative cerebellar disease. neurology 2009; 73:1823-1830

4. Miyai I, Ito M, Hattori N, Mihara M, Hatakenaka M, Yagura H, Sobue G, Nishizawa M. Cerebellar ataxia rehabilitation trial in degenerative cerebellar Neurorehabilitation and neural repair 2012; 26:515-522

5. Ristori G, Romano S, Visconti A, Cannoni S, Spadaro M, Frontali M, Pontieri F, Vanacore N, Salvetti M. Riluzole in cerebellar ataxia A randomized, double-blind, placebo-controlled pilot trial. double-blind, placebo-controlled pilot trial. Neurology 2010; 74:839-845

6. Zesiewicz TA, Greenstein P, Sullivan KL, Wecker L, Miller A, Jahan I, Chen R, Perlman S. A randomized trial of varenicline (Chantix) for the treatment of spinocerebellar ataxia type 3. Neurology 2012; 78:545-550

7. ARGI-U ® Combination Granules Interview Form (EA Pharma Inc.).

8. （In Japanese）戦略的創造研究推進事業CREST研究課題「ポリグルタミン病の包括的治療法の開発」研究終了報告書。研究期間平成21年10月～平成27年3月（研究代表者：貫名　信行）.

9. Minakawa EN, Popiel HA, Tada M, Takahashi T, Yamane H, Saitoh Y, Takahashi Y, Ozawa D, Takeda A, Takeuchi T, Okamoto Y, Yamamoto K, Suzuki M, Fujita H, Ito C,. Yagihara H, Saito Y, Watase K, Adachi H, Katsuno M, Mochizuki H, Shiraki K, Sobue G, Toda T, Wada K, Onodera O, Nagai Y. Arginine is a disease modifier for Brain 2020; *in press (published online ahead of print, 21 May 2020).*

10. Drago F, Continella G, Alloro M, Auditore S, Pennisi G. Behavioural effects of arginine in male rats. pharmacological research communications 1984;. 16:899-907

11. （In Japanese）高木博司. 脳から単離された活性ジペプチド Kyotorphin およびその前駆物質 L-Arginine の生理・薬理作用と慢性痙痛への臨床応用―Kyotorphin 発見 10 年のあゆみ―. 日本薬理学雑誌 1990; 96:85-96

12. Bornhof C, Schwille P, Beijer H, Charbon G. Hemodynamic splanchnic and renal changes associated with the administration of arginine-hydrochloride in dogs.Research in Experimental Medicine 1980; 177:57-70

13. Wang Y-X, Pang CC. Pressor effect of NG-nitro-L-arginine in pentobarbital-anesthetised rats. life sciences 1990; 47:2217-2224

14. M.R.Cernadas, M.J.Gallego, Farré AL, L.Hernando, A.Riesco, S.Grandes, S.Casado, C.Caramelo. l-arginine-induced hypotension. lancet 1990; 336 :1016-1017

15. Rees D, Palmer R, Moncada S. Role of endothelium-derived nitric oxide in the regulation of blood pressure. Proceedings of the National Academy of Sciences 1989; 86:3375-3378. Sciences 1989; 86:3375-3378

16. Schini VB, Vanhoutte PM. L-arginine evokes both endothelium-dependent and -independent relaxations in L-arginine-depleted aortas of the rat. Circulation research 1991; 68:209-216

17. Takeuchi K, Ohuchi T, Kato S, Okabe S. Cytoprotective action of L-arginine against HC1-induced gastric injury in rats: Involvement of nitric oxide? The Japanese Journal of Pharmacology 1993; 61:13-21

18. Thomas G, Farhat MY, Ramwell PW. Effect of L-arginine and substituted arginine compounds on platelet aggregation: role of the endothelium. Thrombosis research 1990; 60:425-429

19. Batlle D, Hays S, Foley R, Chan Y, Arruda JA, Kurtzman NA. proximal renal tubular acidosis and hypophosphatemia induced by arginine. regulation of Phosphate and Mineral Metabolism: Springer; 1982:239-249.

20. （In Japanese）木村成昭, 亀山和人, 細井英司, 斎藤史郎. ラットにおける PGE2, 2-DG および L-arginine の視床下部 GHRH および SRIF の放出に及ぼす影響と GH 分泌との相関. 日本内分泌学会雑誌 1989; 65:113-127

21. Ajinomoto Pharmaceuticals: in-house data (Effects on blood pressure and heart rate in awake healthy rats).

22. Breglia RJ, Ward CO, Jarowski CI. effect of selected amino acids on ethanol toxicity in rats. J Pharm Sci 1973; 62:49-55

23. Ajinomoto Pharmaceuticals: in-house data (oral single-dose toxicity study in juvenile rats).

24. Ajinomoto Pharmaceuticals: in-house data (oral single-dose toxicity study in 6-week-old rats).

25. Ajinomoto Pharmaceuticals: internal data (single intravenous administration and 14-day (12 times) intravenous repeated dose toxicity study in rats).

26. Kadota T, Kondoh H, Chikazawa H, Kuroyanagi K, Ishikawa K, Kawano S, Sakakura K, Takahashi N, Funahashi N, Shimizu N. Cefepime (diHCl/L-arginine blend). blend): intravenous continuous infusion and/or single dose subcutaneous toxicity study in rats and dogs. 612-619

27. Ajinomoto Pharmaceuticals: internal data (1-month oral repeated dose toxicity study and 2-week recovery study in rats).

28. Kaemmerer K, Aly ZH. [The effect of overdoses of amino acids on the rat fetal phase]. Dtsch Tierarztl Wochenschr 1975; 82:457-460

29. Kai S, Kohmura H, Ishikawa K, Kawano S, Sakai A, Kuroyanagi K, Kadota T, Takahashi N. Reproductive and developmental toxicity studies on cefepime K., Kawano K, Ishikawa K, Sakai A, Kuroyanagi K, Kadota T, Takahashi N. Reproductive and developmental toxicity studies on cefepime dihydrochloride administered subcutaneously to rats during the premating, gestation and lactation periods. The Japanese journal of antibiotics 1992 ; 45:642-660

30. Ghosh S, Kundu A, Chattopadhyay K. Small Molecules Attenuate the Interplay between Conformational Fluctuations, Early Oligomerization and Amyloidosis of Alpha Synuclein. Sci Rep 2018; 8:5481

31. Romano S, Coarelli G, Marcotulli C, Leonardi L, Piccolo F, Spadaro M, Frontali M, Ferraldeschi M, Vulpiani MC, Ponzelli F, Salvetti M, Orzi F, Petrucci A, Vanacore N, Casali C, Ristori G. Riluzole in patients with hereditary cerebellar ataxia: a randomised, double-blind, placebo-controlled trial. Lancet Neurol 2015; 14:985-991

32. Low PA, Robertson D, Gilman S, Kaufmann H, Singer W, Biaggioni I, Freeman R, Perlman S, Hauser RA, Cheshire W, Lessig S, Vernino S, Mandrekar J, Dupont WD , Chelimsky T, Galpern WR. Efficacy and safety of rifampicin for multiple system atrophy: a randomised, double-blind, placebo-controlled trial. Lancet Neurol 2014; 13:268-275

33. Poewe W, Seppi K, Fitzer-Attas CJ, Wenning GK, Gilman S, Low PA, Giladi N, Barone P, Sampaio C, Eyal E, Rascol O, Rasagiline-for MSAi. Efficacy of rasagiline in patients with the parkinsonian variant of multiple system atrophy: a randomised, placebo-controlled trial. Lancet Neurol 2015; 14:145-. 152

34. Meier T, Perlman SL, Rummey C, Coppard NJ, Lynch DR. Assessment of the neurological efficacy of idebenone in pediatric patients with Friedreich's ataxia J Neurol 2012; 259:284-291.

35. Yasui K, Yabe I, Yoshida K, Kanai K, Arai K, Ito M, Onodera O, Koyano S, Isozaki E, Sawai S, Adachi Y, Sasaki H, Kuwabara S, Hattori T, Sobue G, Mizusawa H,. Tsuji S, Nishizawa M, Nakashima K. A 3-year cohort study of the natural history of spinocerebellar ataxia type 6 in Japan Orphanet J Rare Dis 2014; 9:118

36. Schmitz-Hubsch T, Fimmers R, Rakowicz M, Rola R, Zdzienicka E, Fancellu R, Mariotti C, Linnemann C, Schols L, Timmann D, Filla A, Salvatore E, Infante J Giunti P, Labrum R, Kremer B, van de Warrenburg BP, Baliko L, Melegh B, Depondt C, Schulz J, du Montcel ST, Klockgether T. Responsiveness of different rating instruments in spinocerebellar ataxia patients Neurology 2010; 74:678-684

37. Jacobi H, Bauer P, Giunti P, Labrum R, Sweeney MG, Charles P, Durr A, Marelli C, Globas C, Linnemann C, Schols L, Rakowicz M, Rola R, Zdzienicka E, Schmitz -Hubsch T, Fancellu R, Mariotti C, Tomasello C, Baliko L, Melegh B, Filla A, Rinaldi C, van de Warrenburg BP, Verstappen CC, Szymanski S, Berciano J,. Infante J, Timmann D, Boesch S, Hering S, Depondt C, Pandolfo M, Kang JS, Ratzka S, Schulz J, Tezenas du Montcel S, Klockgether T. The natural history of spinocerebellar ataxia types 1, 2, 3, and 6: a 2-year follow-up study. neurology 2011; 77:1035-1041

38. Ashizawa T, Figueroa KP, Perlman SL, Gomez CM, Wilmot GR, Schmahmann JD, Ying SH, Zesiewicz TA, Paulson HL, Shakkottai VG, Bushara KO, Kuo SH,. Geschwind MD, Xia G, Mazzoni P, Krischer JP, Cuthbertson D, Holbert AR, Ferguson JH, Pulst SM, Subramony SH. Clinical characteristics of patients with spinocerebellar ataxias 1, 2, 3 and 6 in the US; a prospective observational study. Orphanet J Rare Dis 2013; 8:177
